# GPR65 promotes intestinal mucosal Th1 and Th17 cell differentiation and gut inflammation through downregulating NUAK2

**DOI:** 10.1002/ctm2.771

**Published:** 2022-03-28

**Authors:** Ritian Lin, Wei Wu, Huimin Chen, Han Gao, Xiaohan Wu, Gengfeng Li, Qiong He, Huiying Lu, Mingming Sun, Zhanju Liu

**Affiliations:** ^1^ Center for Inflammatory Bowel Disease Research The Shanghai Tenth People's Hospital Tongji University of School Medicine Shanghai China

**Keywords:** GPR65, inflammatory bowel diseases, Th1 cells, Th17 cells

## Abstract

G protein‐coupled receptor 65 (GPR65), a susceptibility gene for inflammatory bowel diseases (IBD), has been identified to promote Th17 cell pathogenicity and induce T cell apoptosis. However, the potential role of GPR65 in modulating CD4^+^ T cell immune responses in the pathogenesis of IBD stills not entirely understood. Here, we displayed that GPR65 expression was increased in inflamed intestinal mucosa of IBD patients and positively associated with disease activity. It was expressed in CD4^+^ T cells and robustly upregulated through the TNF‐α‐caspase 3/8 signalling pathway. Ectopic expression of GPR65 significantly promoted the differentiation of peripheral blood (PB) CD4^+^ T cells from IBD patients and HC to Th1 and Th17 cells in vitro. Importantly, conditional knockout of *Gpr65* in CD4^+^ T cells ameliorated trinitrobenzene sulfonic acid (TNBS)‐induced acute murine colitis and a chronic colitis in Rag1^–/–^ mice reconstituted with CD45RB^high^CD4^+^ T cells in vivo, characterised by attenuated Th1 and Th17 cell immune response in colon mucosa and decreased infiltration of CD4^+^ T cells, neutrophils and macrophages. RNA‐seq analysis of Gpr65^ΔCD4^ and Gpr65^flx/flx^CD4^+^ T cells revealed that NUAK family kinase 2 (*Nuak2*) acts as a functional target of *Gpr65* to restrict Th1 and Th17 cell immune response. Mechanistically, GPR65 deficiency promoted NUAK2 expression via the cAMP‐PKA‐C‐Raf‐ERK1/2‐LKB1‐mediated signalling pathway. Consistently, silencing of *Nuak2* facilitated the differentiation of Gpr65^ΔCD4^ and Gpr65^flx/flx^CD4^+^ T cells into Th1 and Th17 cells. Therefore, our data point out that GPR65 promotes Th1 and Th17 cell immune response and intestinal mucosal inflammation by suppressing NUAK2 expression, and that targeting GPR65 and NUAK2 in CD4^+^ T cells may represent a novel therapeutic approach for IBD.

## Graphical abstract

1. GPR65 is highly expressed in inflamed intestinal mucosa and mainly expressed in CD4^+^ T cells of IBD patients.
2. GPR65 promotes IBD CD4^+^ T cell differentiation into Th1 and Th17 cells.
3. GPR65 deficiency ameliorates intestinal mucosal inflammation in mice through upregulating NUAK2 expression in CD4^+^ T cells via the cAMP–PKA–C–Raf–ERK1/2‐LKB1‐mediated signalling pathway.

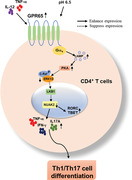

## 1 INTRODUCTION

Inflammatory bowel disease (IBD), comprising Crohn's disease (CD) and ulcerative colitis (UC), is a chronic relapsing inflammatory disorder of the gastrointestinal tract, which is considered to be associated with gene susceptibility, dietary exposure, persistent infection and aberrant immune responses to intestinal microbiota.^1^, ^2^, ^3^, ^4^, ^5^ The family of proton‐sensing G protein‐coupled receptors (GPCRs) encompasses ovarian cancer G protein‐coupled receptor 1 (GPR68), GPR4 and GPR65, which senses extracellular protons.^6^, ^7^ GPR65 is defined originally as T‐cell death‐associated gene 8 (TDAG8),^8^ functions as a proton‐sensing receptor,^9^, ^10^ and is also sensitive to psychosine.^9^, ^11^ It is primarily expressed in T and B lymphocytes as well as in macrophages and other innate immune cells (e.g., neutrophils, eosinophils and mast cells).^6^ Multiple genome‐wide association studies have illustrated that the GPR65 locus is a risk gene for human IBD, arthristis and atopic dermatitis.^12^, ^13^, ^14^, ^15^

Previous study has demonstrated that glucocorticoid‐stimulated T cells highly express GPR65, which further promotes T cell apoptosis.^16^ TDAG8‐deficient mice develop more severe anti‐type II collagen antibody‐induced arthritis,^17^ decrease the survival rate and cardiac function after myocardial infraction,^18^ and promote the LPS‐induced lung injury.^19^ Single‐cell RNA‐seq analysis has confirmed that GPR65 facilitates Th17 cell pathogenicity and that GPR65 deficiency inhibits autoimmune encephalomyelitis (EAE),^20^ whereas another study demonstrated that GPR65 inhibits ongoing autoimmune neuroinflammation in the murine model of EAE and that the absence of iNKT cells mitigates the severity of EAE in CD1d^–/–^Gpr65^gfp/gfp^ mice, indicating that GPR65 in iNKT cells plays a crucial role in restraining EAE.^21^ Besides, mice lacking *Gpr65* have increased susceptibility to *Citrobacter rodentium*‐induced colitis, and epithelial cells (HeLa cells) and macrophages lacking GPR65 exhibit flawed intracellular bacterial clearance and anomalous lysosomes assemblage.^22^ Recent study has illustrated that lack of GPR65 in macrophages exacerbates DSS‐induced acute colitis in mice,^23^ suggesting that GPR65 may participate in the pathogenesis of IBD. Nevertheless, the potential function of GPR65 in regulating mucosal CD4^+^ T cell functions during colitis and maintaining intestinal homeostasis remains not fully understood.

Here, we observed that GPR65 expression level was upregulated in inflamed gut mucosa and peripheral blood (PB)‐CD4^+^ T cells of active IBD patients by contrast with healthy controls (HC), and found that transduction with lentivirus (LV) expressing GPR65 (LV‐GPR65) promoted the differentiation of PB‐CD4^+^ T cells into Th1 and Th17 cells. In comparison with controls, mild colitis was observed in GPR65^ΔCD4^ mice by subjecting to an acute colitis induced by trinitrobenzene sulfonic acid (TNBS) rectally and in a chronic colitis in Rag1^–/–^ mice reconstituted with GPR65^ΔCD4^CD45RB^high^CD4^+^ T cells in vivo, characterised by the attenuated level of proinflammatory cytokines and their transcriptional factors (*Tbx21*, *Rorc*) and decreased invasion of CD4^+^ T cells, neutrophils and macrophages in colon mucosa. RNA‐seq analysis revealed that NUAK family kinase 2 (*Nuak2*) was upregulated in Gpr65^ΔCD4^CD4^+^ T cells and that silencing of *Nuak2* facilitated the differentiation of Gpr65^flx/flx^ and Gpr65^ΔCD4^CD4^+^ T cells into Th1 and Th17 cells. Mechanistically, GPR65 deficiency promoted NUAK2 expression and suppressed Th1 and Th17 cell differentiation via the cAMP‐PKA‐C‐Raf‐ERK1/2‐LKB1‐mediated signalling pathway.

## 2 MATERIALS AND METHODS

### 2.1 Subjects

All patients and healthy donors encompassed in this study were recruited from the Department of Gastroenterology, the Shanghai Tenth People's Hospital of Tongji University (Shanghai, China) from July 2018 to June 2021. The endoscopic specimens were collected from 87 active CD patients (A‐CD), 31 CD patients in remission (R‐CD), 77 active UC patients (A‐UC), 35 UC patients in remission (R‐UC) and 64 HC. Ethylene diamine tetraacetic acid anti‐coagulated peripheral blood samples were collected from 46 A‐CD, 38 R‐CD, 50 A‐UC, 21 R‐UC and 46 HC. The diagnoses of CD and UC were made by combining with clinicopathological characteristics, endoscopic and radiological examination after excluding any autoimmune diseases, pathogen infections and tumors. Furthermore, CD activity index (CDAI), endoscopic score for CD (SES‐CD), Mayo index and UC endoscopic index of severity (UCEIS) were used to evaluate disease severity of CD and UC patients, respectively. The clinical features of IBD patients and healthy individuals are shown in Table S1.

### 2.2 Lentiviral transduction of CD4^+^ T cells

LV expressing GPR65 (LV‐GPR65; transcript ID: NM_003608.4), GPR65 shRNA (LV‐shGPR65; target sequence: ACCCTTTAGAGAAATGGCAAATCAA), and *Nuak2* shRNA (LV‐shNuak2, target sequence: CAGCGTGAATCTGGTTACTACTCCT) were constructed in Hanbio Tech (Shanghai, China). The GPR65 sequence was synthesised as phBLV‐CMV‐MCS‐3Flag‐EF1‐ZSGreen‐T2A‐PURO vector, and GPR65 or Nuak2 siRNA sequence was synthesised into pHBLV‐U6‐MCS‐CMV‐ZsGreen‐PGK‐PURO. *E. coli* strain DH5‐α was used to amplify lentiviral vector and auxiliary packaging vector plasmid (psPAX2 and pMD2G). 293T cells were transfected with the vectors by Lipofiter™, and the supernatant of the virus particles was then collected for further study. The interference efficiency greater than 50% was selected for the shRNA study. CD4^+^ T cells were isolated using anti‐human naive CD4^+^ T cell enrichment set (BD) or anti‐mouse naive CD4^+^ T cell isolation kit (Miltenyi Biotecanti) and stimulated in vitro with anti‐CD3 (5 μg/ml, eBioscience) and anti‐CD28 mAb (2 μg/ml, eBioscience) for 2 days. The pre‐activated cells (1 × 10^5^/well) were transduced with LV expressing GPR65, LV‐shGPR65, LV‐shNuak2 and negative control LV‐NC, respectively. Afterwards, these transfection cells were cultured for 5 days.^24^ Ultimately, cells were collected and performed to identify the transduction efficiency and expression level of different cytokines and related transcription factors by quantitative real‐time PCR (qRT‐PCR). The culture supernatants were gathered to assess cytokine concentration by ELISA.

### 2.3 Mice

CD4‐Cre mice on C57BL/6 background were purchased from Model Organisms Center, Inc (Shanghai, China). Male Rag1^–/–^ mice at 6 weeks old on C57BL/6 background were purchased from the Gem Pharmatech Co., Ltd (Nanjing, China). Rag1^–/–^, Gpr65^flx/flx^, Gpr65^ΔCD4^ and CD4‐Cre mice were reared in the specific pathogen‐free (SPF) animal facility of Tongji University. Eight‐ to 10‐week‐old male mice were applied to experiment following the Guide for the Care and Use of Laboratory Animals.

### 2.4 Construction of Gpr65^ΔCD4^ mice

All mice were C57BL/6 background. Gpr65^flx/–^ mice were constructed at the Shanghai Key Laboratory of Regulatory Biology (Shanghai, China) using the clustered regularly interspaced short palindromic repeats (CRISPR)/Cas9 technique according to *Gpr65* gene sequence (NCBI gene ID: 108682). Gpr65‐201 transcript has two exon regions, and only exon contains a coding region. Therefore, exon2 (1989 bp) was selected as the flox region. Exon 2 of *Gpr65* gene were picked to design sgRNAs: 5′‐ GTTATTGGGCATCGTCTGGTGGG ‐3′, and 5′‐ ATGCCTACCTAAAACTAGGCAGG ‐3′. The genotypes of offspring were identified by PCR (sense primer: 5′‐TCCCTTTCCCTGCTGACA‐3′ and 5′‐GGTCCCTTTCCCTGCTATAACT‐3′; anti‐sense primer: 5′‐TGACAGGGACAGACTGAAGAG‐3′). Gpr65^flx/–^ mice on a C57BL/6J background were mated with CD4‐Cre mice to produce Gpr65^flx/–^CD4‐Cre mice as F1 progeny, and confirmed F1 heterozygotes were intercrossed to obtain Gpr65^ΔCD4^ and Gpr65^flx/flx^ mice for further study.

### 2.5 Trinitrobenzene sulfonic acid‐induced acute mice colitis

The TNBS‐induced colitis mice model was set up as depicted previously.^25^ Male Gpr65^ΔCD4^ and Gpr65^flx/flx^ mice were anaesthetised by intraperitoneal injection 4% chloral hydrate after fasting 24 h. These anaesthetised mice were then slowly implemented intrarectally with 150 ul of 2.5% (w/v) TNBS within 50% ethanol, and the remaining groups were treated with 50% ethanol alone as controls. TNBS or ethanol was administered on day 0, then all mice were immolated for further experiments on day 7 after administration with TNBS or ethanol rectally.

### 2.6 T cell transfer model of chronic colitis in Rag1^–/–^ mice

Splenic CD4^+^ T cells were purified from Gpr65^flx/flx^ and Gpr65^ΔCD4^ mice using anti‐mouse CD4 beads (BD). CD25^−^CD45RB^high^CD4^+^ T cells were separated on a BD FACSAria III, and transferred intraperitoneally into 8 weeks old Rag1^–/–^ mice (0.5 × 10^6^ cells/mouse) by intraperitoneally,^26^ and recipient mice were evaluated for the signs of disease during a period of 7 weeks.^27^, ^28^ All mice were then sacrificed and the severity of colitis was evaluated by H&E staining, flow cytometry and RNA analysis, respectively.

### 2.7 Isolation of lamina propria mononuclear cells

The isolation of lamina propria mononuclear cells (LPMCs) was performed according to our previous study.^24^ LP‐CD4^+^, CD8^+^ T, CD19^+^ B cells were then isolated by immunomagnetic positive selection (BD Biosciences), and dendritic cells (DCs) were isolated by immunomagnetic negative selection (BD Biosciences) following the instructions.

### 2.8 Quantitative real‐time PCR

qRT‐PCR was accomplished as depicted formerly.^29^, ^30^ All primer sequences were listed as indicated in Table S2.

### 2.9 Immunofluorescence staining

Immunofluorescence staining for GPR65, CD4 and NUAK2 expression in the colon tissues was performed as follows: 5‐μm‐thick sections were deparaffinised and rehydrated, followed by antigen retrieval. Normal donkey and goat serum were added by blocking for 1 h, rabbit anti‐human GPR65 or NUAK2 antibody, and rat anti‐human CD4 (diluted at 1:200, Invitrogen) or phosphate buffer saline (PBS) (served as negative control) were then applied in these sections at 4°C all night. Thereafter, these slices were rinsed in PBS, and then treated by Alexa fluor® 488 conjugated goat anti‐rat IgG, Alexa fluor® 594 or Alexa fluor® 488 conjugated donkey anti‐rabbit IgG (1:1000, Invitrogen), respectively, at room temperature for 45 min. Lastly, these sections were stained with Hoechst 33258 and detected by the fluorescence microscope, and negative control group was used to assess the positive cells.

### 2.10 Western blotting analysis

Immunoblotting was performed as depicted formerly.^29^, ^31^ Splenic naïve CD4^+^ T cells were obtained from Gpr65^flx/flx^ and Gpr65^ΔCD4^ mice. The primary antibodies including anti‐GPR65 (1:200, Invitrogen), anti‐PKA (1:1000, Cell Signalling Technologies), anti‐C‐Raf (1:750, Cell Signalling Technologies), anti‐P‐C‐Raf (1:1000, Cell Signalling Technologies), anti‐ERK1/2 (1:500, Invitrogen), anti‐LKB1 (1:500, Cell Signalling Technologies), anti‐NUAK2 (1:500, Invitrogen) and anti‐Actin (1:3000, Santa Cruz Biotechnology) were used. Lastly, the membrane was hatched with HRP‐conjugated secondary antibodies, and then scanned by Amersham Imager 600 ECL system (GE Healthcare).

### 2.11 Statistical analysis

Data were presented as mean ± SEM and analysed using GraphPad Prism 6 software (GraphPad Software Inc). Unpaired Student's *t* tests, multiple *t* test, one‐way analysis of variance (ANOVA), and Tukey's multiple comparison test were performed to assess the significance between different groups. Pearson's correlation was used to analyse the relevance between GPR65 expression in intestinal mucosa and SES‐CD, CDAI, UCEIS, and Mayo score, respectively. Spearman correlation was applied between RNA‐seq data and *Gpr65*. *p *< 0.05 were considered statistically significant.

## 3 RESULTS

### 3.1 GPR65 is highly increased in active IBD and robustly induced in CD4^+^ T cells by TNF‐α

**FIGURE 1:**
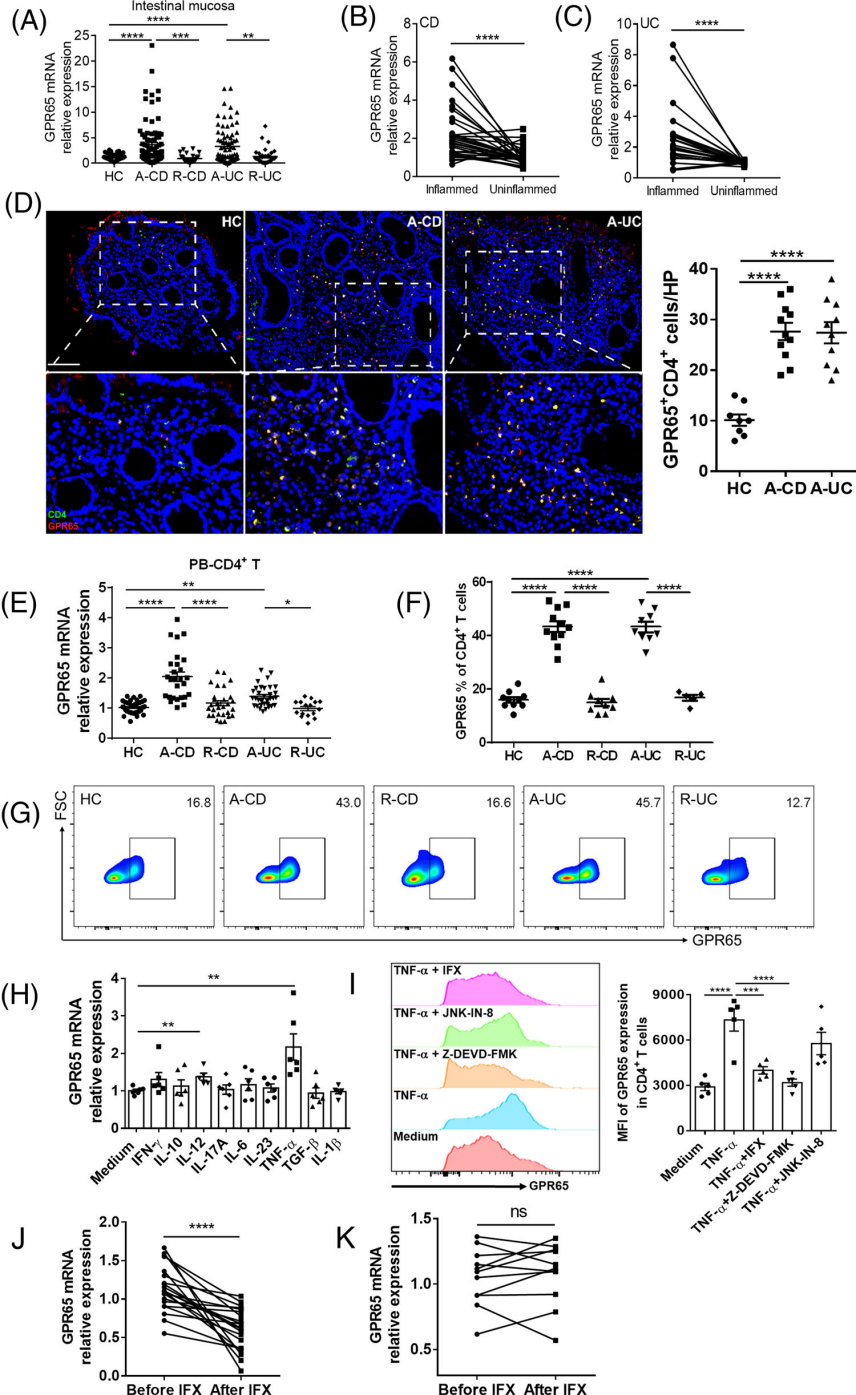
GPR65 is upregulated in active IBD patients and robustly induced in CD4^+^ T cells by TNF‐α. (A) Relative expression of GPR65 mRNA analysed with qRT‐PCR in 87 patients with active CD (A‐CD), 31 CD patients in remission (R‐CD), 77 patients with active UC (A‐UC), 35 UC patients in remission (R‐UC), and 64 healthy controls (HC). (B) Expression of GPR65 mRNA in paired inflamed and uninflamed intestinal tissues of 33 A‐CD patients. (C) Expression of GPR65 mRNA in paired inflamed and uninflamed colon tissues of 25 A‐UC patients. (D) Co‐localisation of GPR65 and CD4^+^ T cells in the colon tissues from 8 HC, 11 A‐CD and 10 A‐UC, respectively, by immunofluorescence staining. Scale bars 100 μm. (E) Expression of GPR65 mRNA in PB‐CD4^+^ T cells from 27 A‐CD, 29 R‐CD, 33 A‐UC, 16 R‐UC and 38 HC. (F and G) GPR65 expression in PB‐CD4^+^ T cells of 10 HC, 12 A‐CD, 9 R‐CD, 9 A‐UC, and 5 R‐UC was analysed by flow cytometry, and the percentages of GPR65^+^ cell proportions in CD4^+^ T cells were shown in the bar chart. (H) PB‐CD4^+^ T cells (5×10^5^/ml) from 6 HC were stimulated with anti‐CD3 mAb (5 μg/ml) and anti‐CD28 mAb (2 μg/ml) in the presence of the indicated cytokines (20 ng/ml) for 72 h, and GPR65 expression was analysed by qRT‐PCR. (I) PB‐CD4^+^ T cells from 5 healthy controls were stimulated in vivo with immobilised anti‐CD3 mAb (5 μg/ml) and soluble anti‐CD28 mAb (2 μg/ml) in the presence of TNF‐α (20 ng/ml) together with anti‐TNF‐α mAb (i.e., infliximab, IFX, 50 ng/ml), JNK inhibitor (JNK‐IN‐8, 1 μM), and caspase‐3/8 inhibitor (Z‐DEVD‐FMK, 50 μM), respectively, for 72 h. GPR65 expression was analysed by flow cytometry, and MFI of GPR65 expression were shown in the bar chart. (J and K) 32 patients with active CD were receiving anti‐TNF mAb (i.e., IFX) treatment at weeks 0, 2 and 6, and intestinal mucosal biopsies were collected from these patients at week 14 after an induction therapy. Twenty‐one patients achieved clinical remission (J), and 11 patients failed to IFX therapy (K). Expression of GPR65 mRNA in intestinal mucosal biopsies was analysed by qRT‐PCR. Data were expressed as mean ± SEM. Statistical analysis was performed using ordinary one‐way analysis of variance (ANOVA) followed by Tukey's multiple comparison test (A, E, F, H and I), and unpaired Student's *t* test (B, C, J and K). *p < 0.05, **p < 0.01, ***p < 0.001, ****p < 0.0001

To clarify whether GPR65 is involved in the etiopathogenesis of IBD, we analysed GPR65 expression level in gut mucosa of IBD patients and the relevance of disease severity. To this purpose, we harvested intestinal biopsies from HC and patients with IBD and analysed its expression by qRT‐PCR and immunofluorescence staining. Figure 1A shows that the mRNA expression of GPR65 was enhanced in inflamed mucosa of active IBD patients by contrast to HC. Interestingly, we found that the mRNA levels of GPR65 were also higher in inflamed mucosa than in the uninflamed mucosa from the same CD or UC patients (Figure 1B,C). Moreover, the numbers of GPR65^+^CD4^+^ cells were significantly increased in inflamed mucosa of active IBD patients in comparison with controls (Figure 1D). We further found that the level of GPR65 mRNA in the intestinal mucosa of CD and UC patients was associated with CDAI and SES‐CD for CD patients, and Mayo index and UCEIS for UC patients, respectively (Figure S1A–D). Additionally, qRT‐PCR and flow cytometric analysis demonstrated that GPR65 expression was also upregulated in PB‐CD4^+^ T cells of active IBD patients in comparison with controls (Figure 1E–G). Collectively, these findings pointed out that GPR65 expression is highly expressed in IBD CD4^+^ T cells, indicating that it may play a key role in the etiopathogenesis of IBD.

Given that lots of proinflammatory factors are existed in the inflamed tissues of IBD, we probed into the fact if these factors contribute to GPR65 expression in CD4^+^ T cells. Towards this end, PB‐CD4^+^ T cells were segregated from HCs and stimulated with immobilised anti‐CD3 mAb and soluble anti‐CD28 mAb in different cytokines in vitro, and expression of GPR65 was determined by qRT‐PCR. Figure 1H demonstrates that TNF‐α and IL‐12 were found to significantly boost GPR65 expression in CD4^+^ T cells. In general, TNF‐α induces T cell activation through different signalling pathways including caspase‐3/8 and JNK. To elucidate the underlying mechanisms by which TNF‐α upregulated the expression of GPR65, we assessed the alterations of GPR65 expression in PB‐CD4^+^ T cells when stimulated in vitro as mentioned above in the presence of TNF‐α together with anti‐TNF‐α mAb (i.e., infliximab, IFX), JNK inhibitor (JNK‐IN‐8), and caspase‐3/8 inhibitor (Z‐DEVD‐FMK), respectively. Intriguingly, we found both IFX and Z‐DEVD‐FMK did significantly suppress TNF‐α‐induced GPR65 expression (Figure 1I). We then detected the expression of GPR65 in gut tissues of active CD patients who received an inductive treatment with IFX and did find that GPR65 expression markedly decreased in gut tissues of active patients with CD in response group (CDAI < 150) at week 14 after the treatment of IFX in contrast to that before the treatment of IFX, but no differences in GPR65 expression were seen in nonresponse group (Figure 1J,K). Therefore, these results suggest that TNF‐α upregulates GPR65 expression in a caspase‐3/8‐dependent manner, and that anti‐TNF‐α treatment suppresses GPR65 expression in CD4^+^ T cells and intestinal mucosa.

To further figure out the divergence of GPR65 expression in different immune cells, we isolated PB‐CD4^+^, CD8^+^ T cells, CD19^+^ B cells and CD14^+^ monocytes from healthy individuals, and determined its expression by qRT‐PCR. Compared with CD14^+^ monocytes, we observed high levels of GPR65 expression in B cells, CD4^+^ and CD8^+^ T cells (Figure S1E). Moreover, GPR65 level was upregulated in LP‐CD4^+^ and CD8^+^ T cells of normal colonic tissues compared to those in DCs (Figure S1F), which were in keeping with earlier report revealing that GPR65 is mainly expressed in T and B lymphocytes.^6^ Moreover, we found that GPR65 mRNA expression was markedly upregulated in CD4^+^ T cells when stimulated with anti‐CD3 and anti‐CD28 mAbs for 48 h (Figure S1G). To further delineate GPR65 expression in different T helper cells, we differentiated naïve PB‐CD4^+^ T cells from healthy donors into different types of cytokines‐secreting effector cells under different conditions for 5 days in vitro, and detected that the level of GPR65 was markedly enhanced in both Th1 and Th17 cells relative to Th0 by qRT‐PCR and flow cytometry (Figure S1H–K). In order to clarify the reason why GPR65 was increased in intestinal mucosal tissues of IBD patients, we collected intestinal mucosal tissues from 10 HC, 14 A‐CD and 12 A‐UC patients, and detected the expression of CD3, CD4, CD8, CD14, IL‐4, IFN‐γ, IL17A and GPR65 mRNA by qRT‐PCR, followed by a correlation analysis (Figure S2A‐H). Notably, we detected that GPR65 expression was only positively relevance with the expression of IFN‐γ and IL17A (Figure S2G,H), implying that an increase of GPR65 mRNA is mainly ascribed to high level of IFN‐γ^+^ and IL17A^+^ cells in inflamed mucosa of IBD patients, which is consistent with the recent report.^32^ These results indicate that GPR65 plays a vital role in orchestrating Th1 and Th17 cell differentiation.

### 3.2 Overexpression of GPR65 in CD4^+^ T cells promotes Th1 and Th17 cell differentiation in IBD patients

**FIGURE 2:**
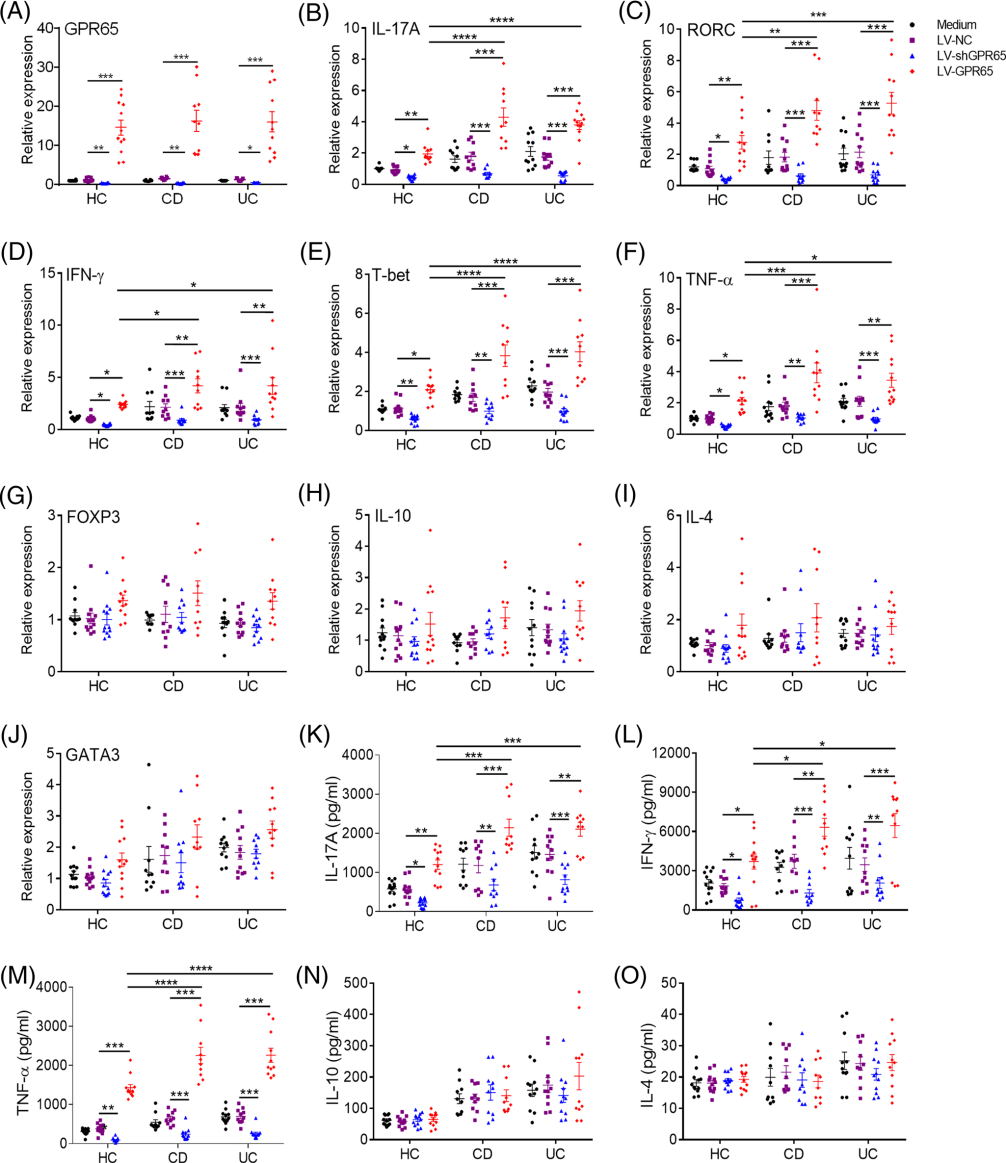
Overexpression of GPR65 in CD4^+^ T cells promotes Th1 and Th17 cell differentiation in IBD patients. (A to O) PB‐CD4^+^ T cells from HC (*n* = 12), A‐CD patients (*n* = 10) and A‐UC patients (*n* = 11) were transfected with lentivirus expressing GPR65 shRNA (LV‐shGPR65), GPR65 (LV‐GPR65) and negative control (LV‐NC), respectively, and then cultured with immobilised anti‐CD3 mAb (5 μg/ml) and soluble anti‐CD28 mAb (2 μg/ml) for 5 days. (A) These transfected CD4^+^ T cells were collected on day 5, and the transduction efficiencies of LV‐shGPR65 and LV‐GPR65 were confirmed by qRT‐PCR. (B to J) The mRNA levels of IL‐17A, RORC, IFN‐γ, TBET, TNF‐α, FOXP3, IL‐10, IL‐4 and GATA3 were determined by qRT‐PCR in these transfected CD4^+^ T cells. (K to O) The culture supernatants of these transfected CD4^+^ T cells were collected for detecting the protein levels of IL‐17A, IFN‐γ, TNF‐α IL‐10, and IL‐4, respectively, using ELISA. Data were expressed as mean ± SEM. Statistical analysis was performed using two‐way analysis of variance (ANOVA) followed by Tukey's multiple comparison test. *p < 0.05, **p < 0.01, ***p < 0.001

Given that the expression level of GPR65 increased in Th1 and Th17 cells, we then investigated whether GPR65 influences the differentiation of IBD CD4^+^ T cell. For this, we transfected PB‐CD4^+^ T cells of HC and active IBD patients with LV expressing GPR65 (LV‐GPR65), GPR65 shRNA (LV‐SHGPR65) and negative control (LV‐NC), respectively. As shown in Figures 2A and S3A, GPR65 expression was increased in LV‐GPR65 transduction CD4^+^ T cells but decreased in LV‐shGPR65 transduction CD4^+^ T cells in comparison with LV‐NC transduction controls. We further detected the level of cytokines and related transcription factors in transfected CD4^+^ T cells. Of note, we detected LV‐GPR65 transduction in IBD CD4^+^ T cells showed higher expression of IL‐17A, RORC, IFN‐γ, TBET, and TNF‐γ compared with controls, whereas LV‐shGPR65 transduction in IBD CD4^+^ T cells leaded to an inverse effect showing a decrease of IL‐17A, RORC, IFN‐γ TBET, and TNF‐α expression (Figure 2B–F). Additionally, neither FOXP3, IL‐10, IL‐4 nor GATA3 expression was influenced in CD4^+^ T cells after LV‐GPR65 or LV‐shGPR65 transduction (Figure 2G–J). Consistently, the quantities of signature cytokines (e.g., IL‐17A, IFN‐γ, TNF‐α, and GM‐CSF) were also upregulated in the supernatants of LV‐GPR65 transduction CD4^+^ T cells but diminished in LV‐shGPR65 transduction CD4^+^ T cells by contrast to controls by ELISA, while IL‐10, IL‐4, IL‐2, and IL‐13 production was not altered (Figures 2K–O, S3B–D). Flow cytometric analysis showed that percentages of IL‐17A^+^ and IFN‐γ^+^

CD4^+^ T cells were upregulated in LV‐GPR65 transduction CD4^+^ T cells compared with controls, whereas LV‐shGPR65 transduction in CD4^+^ T cells resulted in an opposite effect showing a decrease of IL‐17A^+^ and IFN‐γ^+^CD4^+^ T cells (Figure S3E). All together, these data point out that overexpression of GPR65 in CD4^+^ T cells leads to promoting Th1 and Th17 cell differentiation in IBD patients, but lack of GPR65 in CD4^+^ T cells limits this effect.

### 3.3 GPR65 deficiency in CD4^+^ T cells suppresses mice intestinal mucosal inflammation

**FIGURE 3:**
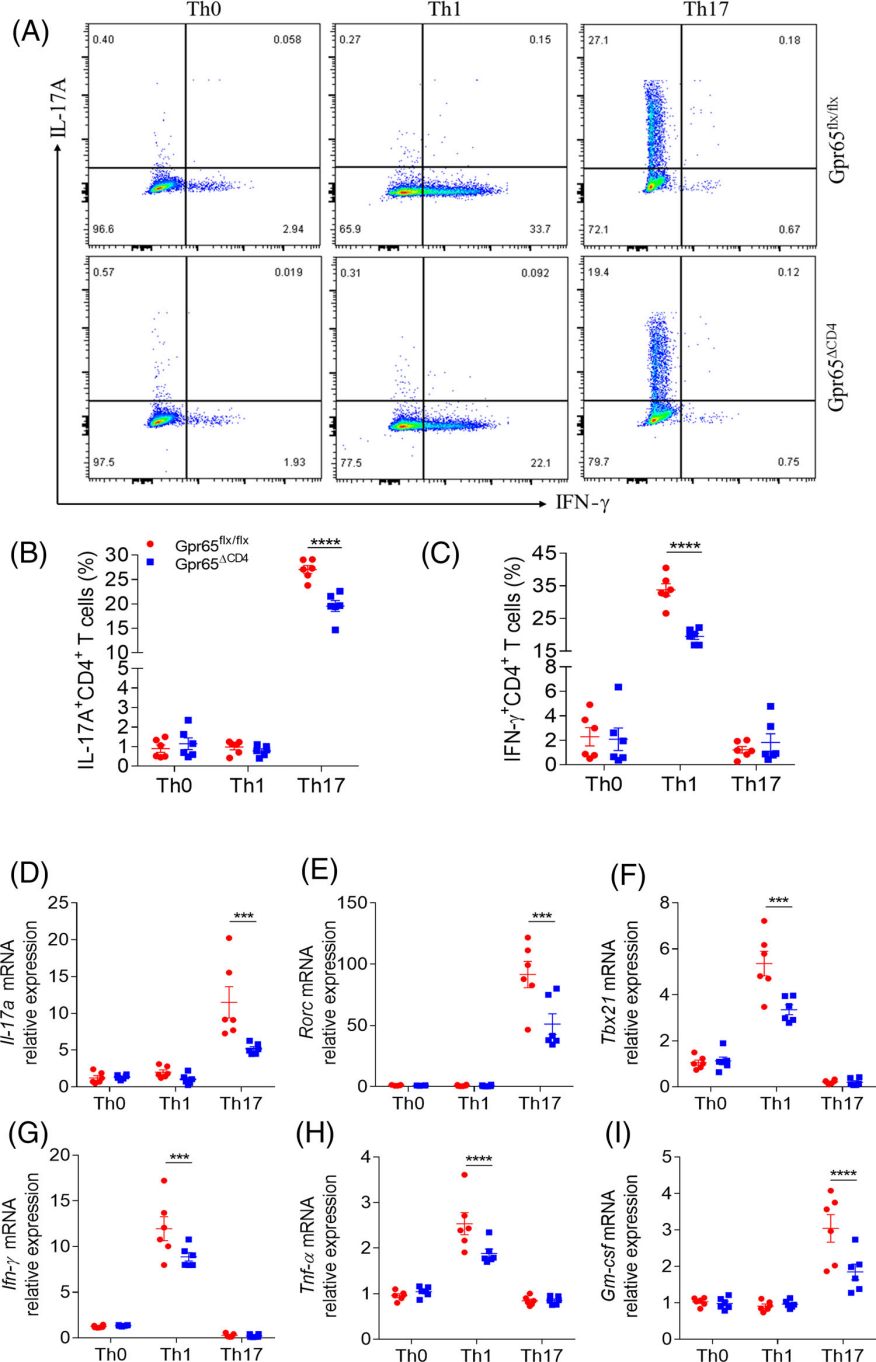
Gpr65^ΔCD4^CD4^+^ T cells compromise Th1 and Th17 cell differentiation in vitro. Splenic naïve CD4^+^ T cells were separated from Gpr65^flx/flx^ and Gpr65^ΔCD4^ mice (*n* = 6/group) using anti‐mouse CD4 magnetic beads, and cultured under different polarising conditions. (A) These polarising CD4^+^ T cells were harvested on day 5, and protein expression of IFN‐γ and IL‐17A was evaluated by flow cytometry. (B and C) Percentages of IFN‐γ ^+^CD4^+^ and IL‐17A^+^CD4^+^ T cells were exhibited in the chart. (D to I) The mRNA levels of *Il‐17a*, *Rorc*, *Ifn‐γ*, *Tbx21, Tnf‐α*, and *Gm‐csf* in these different polarising CD4^+^ T cells were analysed by qRT‐PCR. Data were representative of three independent experiments. Data were presented as mean ± SEM. Statistical analysis was performed with unpaired Student's *t* tests. ***p < 0.001, ****p < 0.0001

For elucidating the character of GPR65 in modulating CD4^+^ T cell differentiation in the etiopathogenesis of mucosal inflammation, we constructed Gpr65^flx/–^ mice by a CRISPR/Cas9 technique, and crossed with CD4‐Cre mice to generate Gpr65^flx/–^CD4‐Cre mice as F1 progeny. Confirmed F1 heterozygotes were then intercrossed to obtain Gpr65^ΔCD4^ and Gpr65^flx/flx^ mice, respectively (Figure S4A–C), which were used for further study. We found that *Gpr65*‐deficiency in CD4^+^ T cells had no effect on the percentage of B cells, CD3^+^, CD4^+^ and CD8^+^ T cells in spleen, MLN and LPMCs compared with those in Gpr65^flx/flx^ littermates (Figure S5A–C). Based on our prior fundings, we raised whether the deficiency of GPR65 could suppress the differentiation of Th1 and Th17 cell. Splenic naïve CD4^+^ T cells were harvested from Gpr65^ΔCD4^ and Gpr65^flx/flx^ mice and cultured in vitro under the Th0, Th1‐ and Th17‐polarising conditions, respectively, for 5 days. Unexpectedly, the percentages of IFN‐γ‐ and IL‐17A‐positive proportions were downregulated in Gpr65^ΔCD4^CD4^+^ T cells under Th1‐ and Th17‐polarising conditions, respectively, in comparison with Gpr65^flx/flx^ controls (Figure 3A–C). Moreover, the level of *Ifn‐*γ, *Tbx21*, *Il‐17a*, *Rorc, Tnf‐α* and *Gm‐csf* was lessened in Gpr65^ΔCD4^CD4^+^ T cells, compared with controls under the same situations by qRT‐PCR (Figure 3D–I).

**FIGURE 4:**
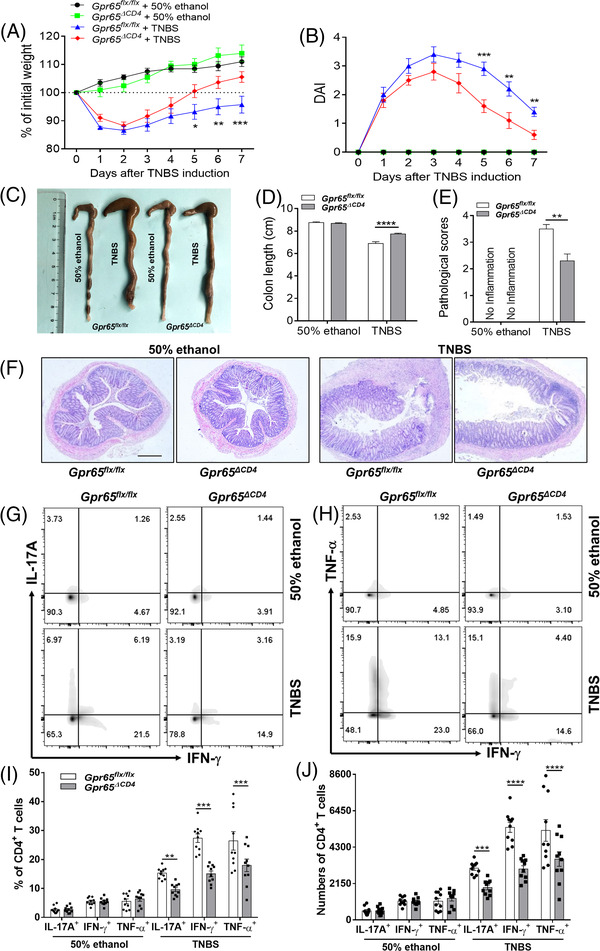
Deficiency of GPR65 in CD4^+^ T cells restrains trinitrobenzene sulfonic acid (TNBS)‐induced acute colitis in mice. TNBS‐induced acute colitis was established in Gpr65^ΔCD4^ and Gpr65^flx/flx^ mice (*n* = 10/group). (A and B) Changes in body weight and disease activity index (DAI) in these mice were recorded daily after TNBS enema. (C and D) Gpr65^ΔCD4^ and Gpr65^flx/flx^ mice were sacrificed on day 7 after administration of TNBS rectally, and colon morphology and length were shown. (E) Histological scores of colonic tissues as indicated. (F) Representative colon sections were stained with H&E. Scale bars 200 μm. (G to J) Intracellular expression of IFN‐γ, IL‐17A and TNF‐α was analysed by flow cytometry in LP‐CD4^+^ T cells after stimulated with PMA and ionomycin for 5 h, the percentages and numbers of IL‐17A^+^, IFN‐γ ^+^, and TNF‐α^+^CD4^+^ T cells were shown in the bar chart. Statistical analyses were performed with unpaired Student's *t* tests. Data were presented as mean ± SEM. Data were representative of three independent experiments. Statistical analysis was performed using Tukey's multiple comparison test (A, B, and D), and multiple *t* test (E, F, I and J). *p < 0.05, **p < 0.01, ***p < 0.001, ****p < 0.0001

To further verify these findings, a TNBS‐induced acute colitis mice model was established in Gpr65^ΔCD4^ and Gpr65^flx/flx^ mice, respectively. We found that Gpr65^ΔCD4^ mice experienced reduced intestinal mucosal inflammation as evidenced by lowered weight loss, downregulated disease activity index (DAI), shortened colon and decreased pathological scores by contrast to those in Gpr65^flx/flx^ littermates (Figure 4A–F). Compared with TNBS‐induced colitic Gpr65^flx/flx^ controls, IL‐17A‐, IFN‐γ‐ and TNF‐α‐expressing CD4^+^ T cells were decreased in lamina propria mononuclear cells (LPMCs) of Gpr65^ΔCD4^ mice by flow cytometric analysis (Figure 4G–J). Consistently, the mRNA level of *Ifn‐*γ, *Tbx21*, *Il‐17a*, *Rorc* and *Tnf‐*α was also reduced in the colonic tissues of TNBS‐induced colitic Gpr6*5^ΔCD4^* mice in comparison with those from Gpr65^flx/flx^ littermates (Figure S6A–E), while no difference in the expression of *Il‐10*, *Foxp3*, *Tgf‐β*, *Il‐4* and *Gata3* was seen between Gpr65^ΔCD4^ and Gpr65^flx/flx^ mice after TNBS enema (Figure S6F–J).

**FIGURE 5:**
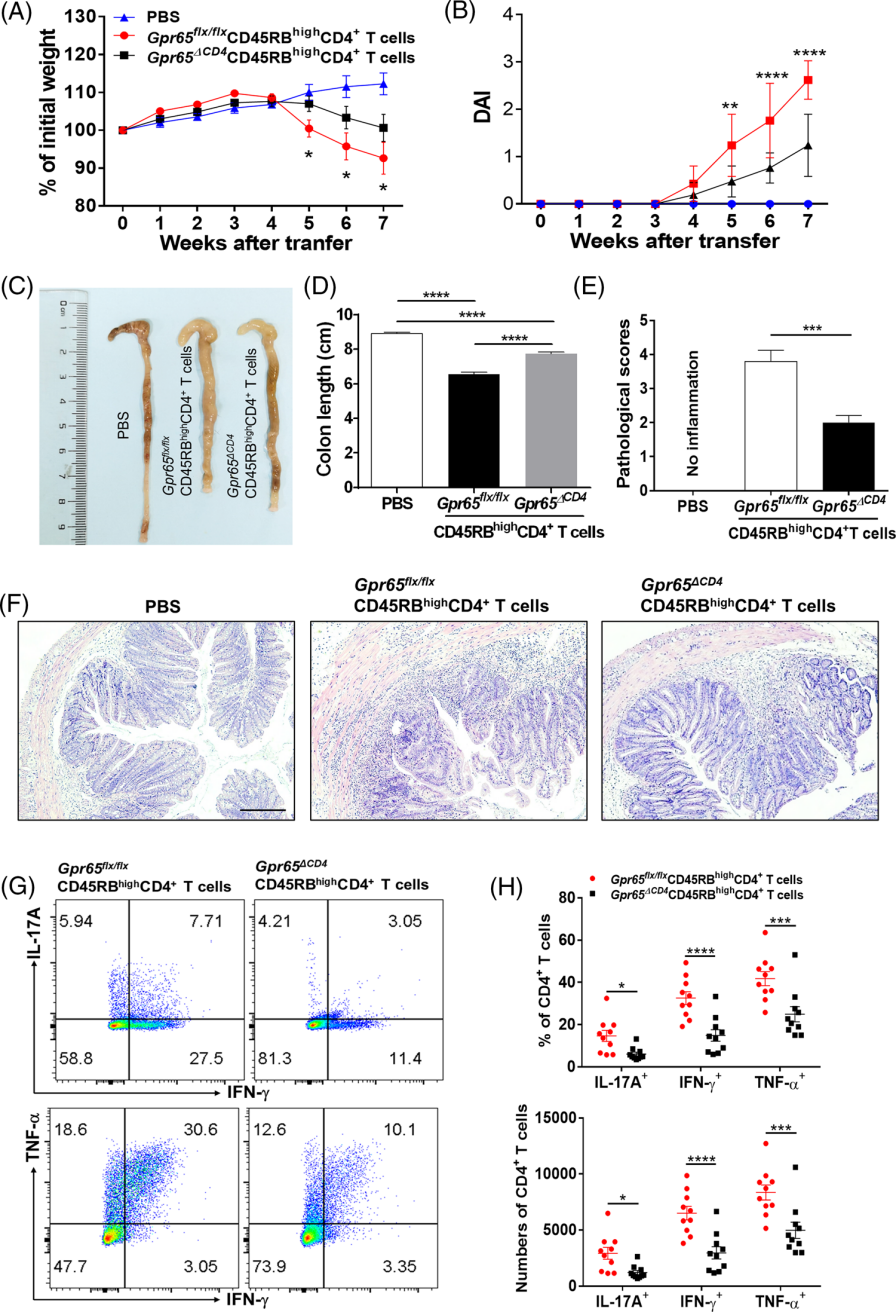
Gpr65^ΔCD4^CD45RB^high^CD4^+^ T cells mitigate chronic colitis in Rag1^–/–^ mice. Splenic CD25^−^CD45RB^high^CD4^+^ T cells were isolated from Gpr65^flx/flx^ and Gpr65^ΔCD4^ mice (*n* = 5/group) by utilising flow cytometry, and infected intraperitoneally into 8‐week‐old Rag1^–/–^ mice (5 × 10^5^ cells/mouse, *n* = 10/group). (A) Mice were weighed weekly after T cell transfer, and changes in body weight were seen in Rag1^–/–^ mice. (B) Disease activity index (DAI) were calculated daily after T cell transfer. (C and D) Mice were sacrificed on week 7 after T cell transfer, and colon morphology and length were shown. (E and F) Calculation of pathological score of the colonic tissues, and representative colon sections were stained with H&E. Scale bars 100 μm. (G) Lamina propria mononuclear cells (LPMCs) were harvested from the colons of Rag1^–/–^ mice transferred with Gpr65^flx/flx^ and Gpr65^ΔCD4^CD45RB^high^CD4^+^ T cells, respectively, and intracellular expression of IL‐17A, IFN‐γ, and TNF‐α in LP‐CD4^+^ T cells was analysed by flow cytometry. (H) Percentages and numbers of IL‐17A^+^CD4^+^, IFN‐ γ ^+^CD4^+^ and TNF‐α^+^CD4^+^ T cells were shown in the chart. Statistical analyses were performed with unpaired Student's *t* tests. Data were presented as mean ± SEM. Data were representative of three independent experiments. Statistical analysis was performed using Tukey's multiple comparison test (A, B and D), and unpaired Student's *t* test (E and H). *p < 0.05, **p < 0.01, ***p < 0.001, ****p < 0.0001

Additionally, we performed a complementary experiment and explored whether deficiency of *Gpr65* in CD4^+^ T cells affected the development of chronic colitis in mice. We adoptively transferred CD45RB^high^CD4^+^ T cells from Gpr65^ΔCD4^ or Gpr65^flx/flx^ mice into Rag1^–/–^ recipients intraperitoneally.^26^ Consequently, we found that Rag1^–/–^ mice transferred with Gpr65^ΔCD4^CD45RB^high^CD4^+^ T cells displayed lowered weight loss, decreased DAI, less shortened colon, and decreased inflammatory cell infiltrations (e.g., CD4^+^ T cells and F4/80^+^ macrophages) in inflamed colonic tissues by contrast to the recipient mice reconstituted with CD45RB^high^CD4^+^ T cells from Gpr65^flx/flx^ littermates (Figure 5A–F, and Figure S7). Flow cytometric analysis further revealed lower proportions of IL‐17A^+^‐, IFN‐γ^+^‐ and TNF‐α^+^‐expressing CD4^+^ T cells from Rag1^–/–^ mice reconstituted with Gpr65^ΔCD^ *^4^* CD45RB^high^CD4^+^ T cells by contrast to controls (Figure 5G,H). Consistent with these results, the mRNA levels of *Ifn‐*γ, *Tbx21*, *Tnf‐*α, *Il‐17a* and *Rorc* were also downregulated in the colonic tissues of Gpr65^ΔCD4^CD45RB^high^CD4^+^ T cell‐reconstituted Rag1^–/–^ mice relative to those from controls, but no statistical difference was observed in the mRNA level of *Il‐10*, *Il‐4* and *Gata3* between these two groups (Figure S8A–H). Overall, these data reveal that the lack of *Gpr65* in CD4^+^ T cells could restrain intestinal mucosal inflammation via diminishing Th1 and Th17 cell immune response.

### 3.4 GPR65 deficiency promotes expression of NUAK2

**FIGURE 6:**
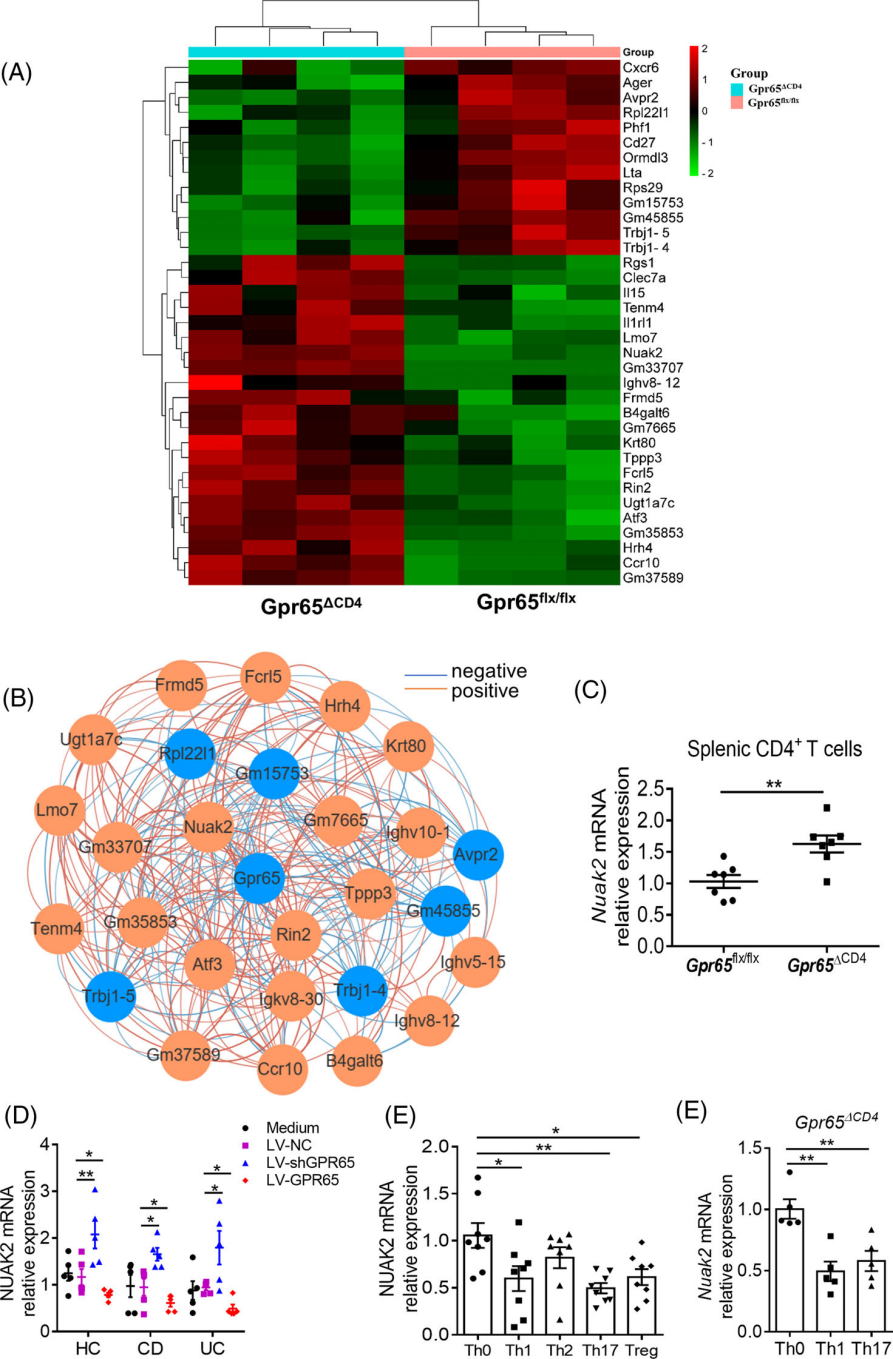
GPR65 deficiency promotes NUAK2 expression in CD4^+^ T cells. Naïve splenic CD4^+^ T cells (*n* = 5/group) were harvested from Gpr65^flx/flx^ and Gpr65^ΔCD4^ mice for RNA‐seq analysis. (A) Heatmap shows the differentially expressed genes between Gpr65^flx/flx^ and Gpr65^ΔCD4^CD4^+^ T cells. (B) Differentially expressed genes with a Spearman correlation coefficient greater than 0.8 with *Gpr65* in RNA‐seq data. The blue colour indicates a decrease of gene expression, and the orange colour indicates an increase of gene expression in Gpr65^ΔCD4^CD4^+^ T cells. (C) Expression of *Nuak2* mRNA was detected in Gpr65^flx/flx^ and Gpr65^ΔCD4^CD4^+^ T cells (*n* = 7/group) by qRT‐PCR. (D) Expression of *Nuak2* mRNA was determined in LV‐shGPR65 and LV‐GPR65 transfected PB‐CD4^+^ T cells from HC, A‐CD, and A‐UC (*n* = 5/group), respectively. (E) PB‐CD4^+^ T cells were isolated from 8 healthy individuals and cultured under Th0‐, Th1‐, Th2‐, Th17‐ and Treg‐polarising conditions, respectively, for 5 days, and expression of *Nuak2* mRNA was assessed by qRT‐PCR. (F) Naïve splenic CD4^+^ T cells were isolated from Gpr65^ΔCD4^ mice and cultured under Th0‐, Th1‐ and Th17‐ polarising conditions, respectively, for 5 days, and expression of *Nuak2* mRNA was assessed by qRT‐PCR. Data were expressed as mean ± SEM. Statistical analysis was performed using Spearman correlation (B), unpaired Student's *t* test (C), two‐way analysis of variance (ANOVA) followed by Tukey's multiple comparison test (D), and ordinary one‐way ANOVA (E, F). *p < 0.05, **p < 0.01

As *Gpr65* deletion in CD4^+^ T cells could suppress Th1 and Th17 cellular immune response and ameliorate intestinal mucosal inflammation. We next dissected the potential mechanisms whereby *Gpr65* confines colitis and regulates the differentiation of CD4^+^ T cell. In this respect, naïve CD4^+^ T cells were sorted from spleen of Gpr65^ΔCD4^ and Gpr65^flx/flx^ mice, and then RNA‐seq analysis was performed to decipher the distinct gene expression. Figure 6A shows the decreased expression of proinflammatory genes, such as *Cxcr6*,^33^ *Ager*,^34^ *Cd27*,^35^ and IBD‐associated genetic risk factor *Ormdl3* ^36^ in CD4^+^ T cells of Gpr65^ΔCD4^ mice. Interestingly, we observed that *Nuak2*, which belongs to the AMP‐activated protein kinase family,^37^ was increased in CD4^+^ T cells of Gpr65^ΔCD4^ mice and negatively correlated with *Gpr65* expression (Figure 6A–C). Previous studies have shown that adipocyte‐specific ablation of *Nuak2* promotes adipose inflammation, and that NUAK2 mutation in humans causes anencephaly.^38^, ^39^ Intriguingly, we found that NUAK2 expression was upregulated in LV‐shGPR65 transduction PB‐CD4^+^ T cells but reduced in LV‐GPR65 transduction PB‐CD4^+^ T cells relative to LV‐NC controls in IBD patients (Figure 6D). The mRNA levels of NUAK2 were substantially decreased in Th1, Th17 and Treg cells compared with those in Th0 cells (Figure 6E), and the frequencies of NUAK2^+^IFN‐γ^+^ and NUAK2^+^IL17A^+^ cell proportions were decreased in Th1 and Th17 cells, respectively, compared with those in Th0 cells in healthy donors (Figure S9A,B), suggesting NUAK2 plays a key role in Th1 and Th17 cell differentiation. Similarly, the expression of Nuak2 mRNA was also decreased in Th1 and Th17 cells than in Th0 cells in splenic CD4^+^ T cells of Gpr65^ΔCD4^ mice (Figure 6F). Additionally, we observed that acidic pH (6.5) could promote cAMP production in the splenic CD4^+^ T cells of Gpr65^flx/flx^ littermates. However, a tendency to an increase in the levels of cAMP was detected in Gpr65^∆CD4^CD4^+^ T cells, but did not reach a significant difference (Figure S9C), implying that acidic pH (6.5) increases cAMP production in CD4^+^ T cells in a GPR65‐dependent way. Therefore, these results point out that GPR65 modulates Th1 and Th17 cell differentiation via a NUAK2‐dependent pathway.

**FIGURE 7:**
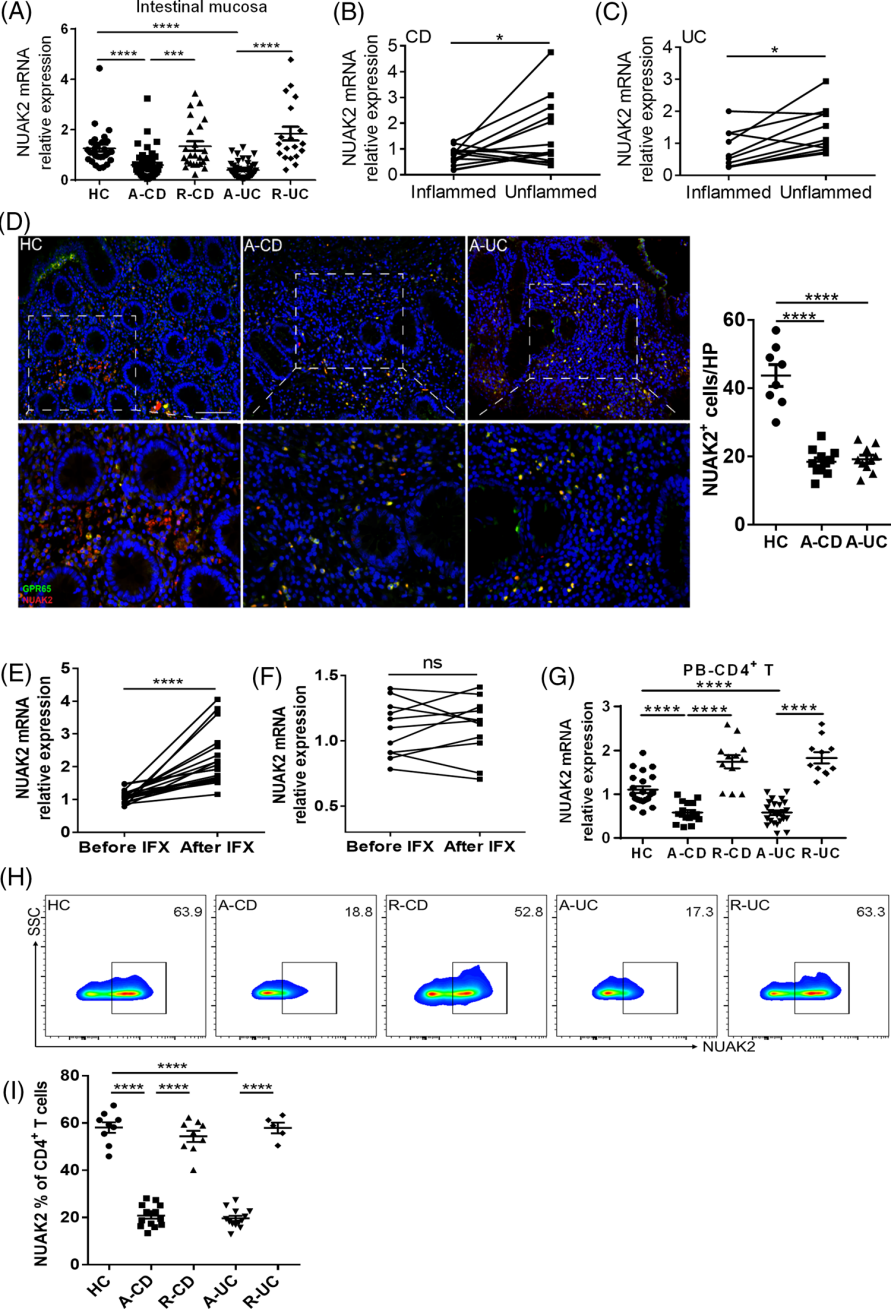
NUAK2 is reduced in active IBD patients. (A) Expression of NUAK2 mRNA levels was analysed by qRT‐PCR in the intestinal mucosa of 44 patients with A‐CD, 24 patients with R‐CD, 43 patients with A‐UC, 19 patients with R‐UC and 28 HC, respectively. (B) Expression of NUAK2 mRNA in paired inflamed and uninflamed intestinal tissues of 14 A‐CD patients. (C) Expression of NUAK2 mRNA in paired inflamed and uninflamed colon tissues of 11 A‐UC patients. (D) Co‐localisation of NUAK2 and GPR65 in intestinal tissues from 8 HC, 11 A‐CD and 10 A‐UC, respectively, by immunofluorescence staining. Scale bars 100 μm. (E and F) 32 patients with active CD were receiving anti‐TNF mAb (i.e., infliximab, IFX) treatment at weeks 0, 2 and 6, and intestinal mucosal biopsies were collected from these patients at week 14 after an induction therapy. Twenty‐one patients achieved clinical remission (E), and 11 patients failed to IFX therapy (F). Expression of NUAK2 mRNA in intestinal mucosal biopsies was analysed by qRT‐PCR. (G) Expression of NUAK2 mRNA in PB‐CD4^+^ T cells from 17 A‐CD, 12 R‐CD, 25 A‐UC, 11 R‐UC and 22 HC, respectively. (H) NUAK2 expression in PB‐CD4^+^ T cells of 9 HC, 14 A‐CD, 9 R‐CD, 13 A‐UC and 5 R‐UC was analysed by flow cytometry, and percentages of NUAK2^+^ cell proportions of CD4^+^ T cells were shown in the bar chart (I). Data were expressed as mean ± SEM. Statistical analysis was performed using ordinary one‐way analysis of variance (ANOVA) (A, G, and I), unpaired Student's *t* test (B, C, E and F). *p < 0.05, **p < 0.01, ***p < 0.001, ****p < 0.0001

Subsequently, we determined NUAK2 expression in intestinal mucosal tissues of IBD patients and healthy individuals by qRT‐PCR and immunofluorescence. We detected that the mRNA level of NUAK2 was diminished in inflamed gut mucosa of active IBD patients by contrast to controls (Figure 7A), and that the same patterns were present in inflamed mucosa relative to the uninflamed mucosa tissues of the same CD or UC patients (Figure 7B,C). The protein levels of NUAK2 were also diminished in inflamed gut mucosa from active IBD patients by contrast to controls (Figure 7D). Besides, we also measured NUAK2 mRNA level in gut mucosa of active CD patients who received an inductive treatment with IFX and found that NUAK2 mRNA level was upregulated in gut mucosa of active CD patients in Response group (CDAI < 150) at week 14 after IFX therapy by contrast to that before IFX therapy, but there was not altered in the nonresponse group (Figure 7E,F). In line with these data, the NUAK2 expression was also diminished in PB‐CD4^+^ T cells of patients with IBD by qRT‐PCR and flow cytometric analysis (Figure 7G–I). Collectively, these results reveal that NUAK2 is negatively correlated with GPR65 expression and functions as regulating the differentiation of CD4^+^ T cells in gut mucosa.

### 3.5 GPR65 deficiency suppresses Th1 and Th17 cell immune response via the C‐Raf‐EKR1/2‐LKB1‐NUAK2 signalling pathway

**FIGURE 8:**
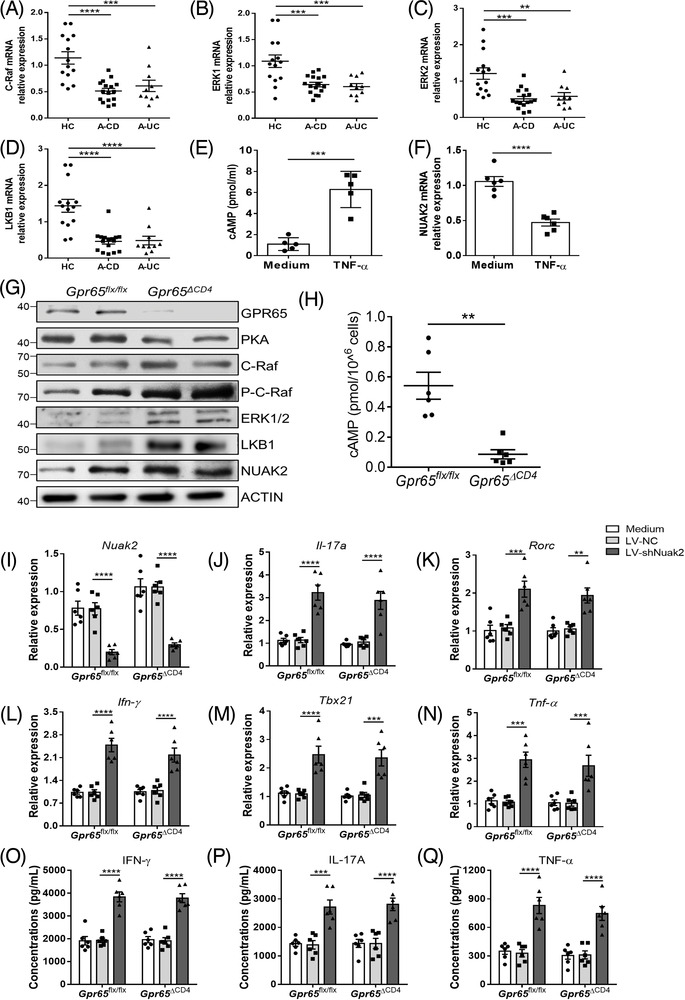
GPR65 deficiency suppresses Th1 and Th17 cell immune response via activating the C‐Raf‐EKR1/2‐LKB1‐NUAK2 pathway in CD4^+^ T cells. (A to D) The expression of CRAF, ERK1/2, and LKB1 in PB‐CD4^+^ T cells from 14 HC, 16 A‐CD, and 10 A‐UC was detected by qRT‐PCR. (E and F) PB‐CD4^+^ T cells from five healthy controls were stimulated in vivo with immobilised anti‐CD3 mAb (5 μg/ml) and soluble anti‐CD28 mAb (2 μg/ml) in the presence of TNF‐α (20 ng/ml) for 72 h. cAMP level was analysed by ELISA, and NUAK2 expression was determined by qRT‐PCR. (G) Representative protein levels of GPR65, PKA, C‐Raf, P‐C‐Raf, ERK1/2, LKB1, and NUAK2 were detected in Gpr65^flx/flx^ and Gpr65^ΔCD4^ splenic CD4^+^ T cells by Western blotting. (H) Production of cAMP in splenic CD4^+^ T cells of Gpr65^flx/flx^ and Gpr65^ΔCD4^ mice was assessed by ELISA (*n* = 6/group). (I to Q) Splenic CD4^+^ T cells of Gpr65^flx/flx^ and Gpr65^ΔCD4^ mice (*n* = 6/group) were transfected with lentivirus expressing *Nuak2* shRNA and negative control (LV–NC), respectively, and then cultured with immobilised anti‐CD3 mAb (5 μg/ml) and soluble anti‐CD28 mAb (2 μg/ml) for 5 days. (I) The transduction efficiency of LV‐shNuak2 was confirmed by qRT‐PCR. (J to N) The mRNA levels of *Il‐17a*, *Rorc*, *Ifn‐γ*, *Tbx21*, and *Tnf‐α* were determined by qRT‐PCR in these transfected CD4^+^ T cells. (O to Q) The culture supernatants of these transfected CD4^+^ T cells were also harvested to determine the protein levels of IL‐17A, IFN‐γ, and TNF‐α, respectively, by ELISA. Data were expressed as mean ± SEM. Statistical analysis was performed using one‐way ANOVA (A to D), unpaired Student's *t* test (E, F and H), two‐way ANOVA followed by Tukey's multiple comparison test (I to Q). *p < 0.05, **p < 0.01, ***p < 0.001, ****p < 0.0001

Previous reports have demonstrated that the Gα_s_‐mediated cAMP‐PKA signalling axis restrains the C‐Raf‐ERK1/2 signal path in mitogen‐activated protein kinase (MAPK) networks by regulating the differentiation, proliferation and apoptosis of cells,^40^, ^41^ and that serine/threonine kinase 11 (LKB1), a rate‐limiting enzyme, is also associated with MAPK to stimulate NUAK2.^37^ We then sought to investigate whether GPR65 as an activator of Gα_s_ could regulate NUAK2 expression via the cAMP‐PKA‐C‐Raf‐ERK1/2‐LKB1 signalling pathway. We detected the expression of CRAF, ERK1/2, and LKB1 in PB‐CD4^+^ T cells of IBD patients and healthy donors by qRT‐PCR, and observed that the expression of CRAF, ERK1/2, and LKB1 was downregulated in IBD PB‐CD4^+^ T cells (Figure 8A–D). Additionally, we found that the level of cAMP was significantly increased but NUAK2 expression was markedly decreased in PB‐CD4^+^ T cells when stimulated with TNF‐α in vitro for 72 h (Figure 8E,F). Combined with the previous findings, GPR65 expression was observed to be increased after TNF‐α stimulation (Figure 1H), suggesting that TNF‐α powerfully facilitates GPR65 expression, followed by upregulated activation of cAMP and inhibition of NUAK2 expression. To further corroborate this statement, we obtained splenic CD4^+^ T cells from Gpr65^flx/flx^ and Gpr65^ΔCD4^ mice and then detected cAMP level and the protein expression of PKA, C‐Raf, P‐C‐Raf, ERK1/2, LKB1, and NUAK2 by ELISA and Western blotting, respectively. Unexpectedly, we found that the production of cAMP and PKA was reduced, while the protein levels of C‐Raf, P‐C‐Raf, ERK1/2, LKB1 and NUAK2 were upregulated in Gpr65^ΔCD4^CD4^+^ T cells compared with controls (Figures 8G,H and S9D–I).

To elucidate whether GPR65 deficiency suppressed Th1 and Th17 cell immune response through an increase of NUAK2 expression, we cultured splenic naïve CD4^+^ T cells of Gpr65^flx/flx^ and Gpr65^ΔCD4^ mice, and then transfected with LV‐shNuak2 for 5 days in vitro to assess the levels of Th1‐ and Th17‐related cytokines by qRT‐PCR and ELISA, respectively. As presented in Figure 8I, the mRNA levels of *Nuak2* were found to be decreased in LV‐shNuak2‐transfected Gpr65^flx/flx^ and Gpr65^ΔCD4^CD4^+^ T cells compared to LV‐NC‐transfected controls. Of note, we found that LV‐shNuak2‐transfected Gpr65^flx/flx^ and Gpr65^ΔCD4^CD4^+^ T cells exhibited higher levels of *Il‐17a*, *Rorc*, *Ifn‐γ*, *Tbx21*, and *Tnf‐α* mRNA and higher frequencies of IL‐17A^+^ and IFN‐*γ* ^+^CD4^+^ T cells compared with controls (Figures 8J–N and S10A–C). Similarly, concentrations of IL‐17A, IFN‐*γ*, and TNF‐*α* were also upregulated in the supernatants of LV‐shNuak2 transduction of Gpr65^flx/flx^ and Gpr65^ΔCD4^CD4^+^ T cells compared with controls by ELISA (Figure 8O–Q). All together, these results point out that lack of *Nuak2* in Gpr65^ΔCD4^CD4^+^ T cells restores the ability to differentiate into Th1 and Th17 cells.

## 4 DISCUSSION

Profound infiltration of activated colitogenic immune cells and dysregulated CD4^+^ T cell‐mediated immune responses in the intestinal mucosa are the pathological characteristics of IBD,^3^, ^42^ while the underlying mechanisms whereby these immune cells are ongoing activation in the gut mucosa are still elusive. Although genetic variants in GPR65 locus are associated with the development of IBD,^12^, ^13^ how GPR65 regulates CD4^+^ T cell differentiation in the intestinal mucosa during IBD remains undefined. Here, we unravelled that the expression of GPR65 was enhanced in inflamed gut mucosa and PB‐CD4^+^ T cells of active IBD patients and identified that GPR65 could facilitate Th1 and Th17 cell differentiation and participate in the etiopathogenesis of colitis by impeding NUAK2 expression.

GPR65 as a susceptibility gene for human IBD^12^, ^13^ is originally identified the function in the activation‐induced apoptosis of T cells,^8^ indicating that it may regulate T cell differentiation and contribute to the pathogenesis of IBD. In this paper, we detected that GPR65 was increased in inflamed gut mucosa and PB‐CD4^+^ T cells of active IBD patients and closely associated with disease activity. We also found that TNF‐α could markedly boost GPR65 expression in PB‐CD4^+^ T cells in a caspase‐3/8‐dependent manner. These data were consistent with previous report showing that glucocorticoid could induce GPR65 expression in CD4^+^CD8^+^ double‐positive thymocytes and then promote apoptosis through the caspase‐3, ‐8 and ‐9‐mediated pathway.^16^ Moreover, we observed higher levels of GPR65 mRNA in Th1 and Th17 cells than cultured in Th0 cells, suggesting that GPR65 could regulate the differentiation function of effector CD4^+^ T cells under inflammatory conditions. These findings allowed us to further investigate the potential function of GPR65 on the differentiation of IBD CD4^+^ T cell, we then performed lentiviral transduction experiments in vitro and did find that LV‐GPR65 transduction IBD CD4^+^ T cells preferentially differentiated into Th1 and Th17 cells, while LV‐shGPR65 transduction CD4^+^ T cells displayed opposite effects. Moreover, we also found that GM‐CSF expression was upregulated in the supernatants of LV‐GPR65 transduction CD4^+^ T cells but diminished in those of LV‐shGPR65 transduction CD4^+^ T cells compared with controls, which is consistent with previous report.^43^ Therefore, our data suggest that GPR65 promotes Th1 and Th17 cell immune response, in agreement with previous study demonstrating that GPR65 promotes Th17 cell‐associated pathogenicity.^20^

Accumulating lines of evidence have shown that GPR68, GPR4 and GPR65 belong to the family of proton‐activated GPCRs, which are activated at pH 6.8 and play an important role in pH homeostasis.^44^, ^45^ In addition to promoting T cell apoptosis in vitro, an adoptive transfer of Gpr65^–/–^CD4^+^ T cells into Rag1^–/–^ mice could prevent from EAE, suggesting that GPR65 augments encephalitogenic T cell generation.^20^ Moreover, deletion of GPR68 or GPR4 ameliorates gut mucosal inflammation in mouse model of colitis,^46^, ^47^, ^48^ indicating these proton‐activated GPCRs may act as a proinflammatory role in the development of colitis. But other researches demonstrated that the lack of GPR65 in macrophages and epithelial cells exhibits a detrimental role in colitis due to lysosomal dysfunction and impaired clearance of intracellular bacteria.^22^, ^23^ To clarify whether GPR65 modulates mucosal CD4^+^ T cell immune responses and promotes the development of colitis, we constructed mice with CD4^+^ T cell‐specific knockout of *Gpr65* gene (Gpr65^ΔCD4^ mice) and investigated the potential role of GPR65 in modulating CD4^+^ T cell activation during colitis. Consequently, we found that Gpr65^ΔCD4^CD4^+^ T cells decreased the expression of IFN‐γ and IL‐17A under Th1 and Th17 differentiation situations, respectively, by contrast to controls. Importantly, we utilised both acute and chronic colitis models and observed that the lack of *Gpr65* in CD4^+^ T cells could alleviate intestinal mucosal inflammation as evidenced by lowered weight loss, decreased DAI, less shortened colon, and decreased infiltration of IFN‐γ^+^, IL‐17A^+^ and TNF‐α^+^CD4^+^ T cells, suggesting that lack of *Gpr65* in CD4^+^ T cells restrains intestinal mucosal inflammation. However, our results were different from the published data showing that more severe colitis was observed in Gpr65^–/–^ mice induced by DSS and *Citrobacter rodentium*, characterised by the increase of proinflammatory mediators (e.g., *Ifn‐γ*, *Tnf* and *iNOS*) released by macrophages and lysosomal dysfunction of epithelial cells.^22^, ^23^ Moreover, the recent studies found that GPR65 deficiency aggravates LPS‐induced lung injury and atopic dermatitis in mouse models.^15^, ^19^ Another study found that GPR65 can inhibit intestinal inflammation and colitis‐associated colorectal cancer development.^32^ These differences may be ascribed to different biological backgrounds, disease models, mouse strains, microbiomes and potential effects of GPR65 on different immune cells.

Gα_s_‐mediated stimulation substantially inhibits MAPK‐associated molecules such as ERK1/2, ERK5 and MEK5 through the cAMP‐PKA‐dependent pathway,^40^ and GPR65 functions as a subunit of Gα_s_ to induce cAMP production. We also substantiated that the levels of cAMP and PKA were reduced, while the levels of C‐Raf, ERK1/2, LKB1 and NUAK2 were upregulated in Gpr65^ΔCD4^CD4^+^ T cells. The previous study has implicated that *Nuak2* could repress inflammation in adipocyte.^38^ We observed an increase of *Nuak2* expression in Gpr65^ΔCD4^CD4^+^ T cells but a decrease in Th1 and Th17 cells, and that LV‐shNuak2‐transfected Gpr65^flx/flx^ and Gpr65^ΔCD4^CD4^+^ T cells could facilitate Th1 and Th17 cell immune response, suggesting a critical role of *Nuak2* in inhibiting Th1 and Th17 cell differentiation. Our data were consistent with previous study showing that Gnas^ΔCD4^CD4^+^ T cells, being deficient in Gα_s_ and cAMP, display limited inflammatory responses and impair the differentiation into Th17 and Th1 cells.^49^ Importantly, we unveiled a distinct mechanism, whereby GPR65 orchestrates Th1 and Th17 cell differentiation by suppressing NUAK2 expression in intestinal mucosa.

In summary, our study has demonstrated that GPR65 functions as a critical contributor to the pathogenesis of IBD and that that blocking GPR65 in CD4^+^ T cells appears to downregulate the immune response of Th1 and Th17 cell via the cAMP‐PKA‐C‐Raf‐ERK1/2‐LKB1‐NUAK2 signalling pathway, leading to alleviating intestinal mucosal inflammation. Therefore, these data have shed some light on better understanding the pathogenesis of IBD and help us develop novel strategies on targeting GPR65 and NUAK2 in mucosal CD4^+^ T cells for the treatment of IBD.

## CONFLICT OF INTEREST

The authors declare that there is no conflict of interest that could be perceived as prejudicing the impartiality of the research reported.

## Supporting information

Supporting information

Supporting information
