# Supplementary material for: GPR65 promotes intestinal mucosal Th1 and Th17 cell differentiation and gut inflammation through downregulating NUAK2
Source: Clin Transl Med. 2022 Mar 28;12(3):e771. doi: 10.1002/ctm2.771 (PMC8958354; doi:10.1002/ctm2.771)
Supplement: Supplementary file 1 — Supporting information [file CTM2-12-e771-s001.docx]

**SUPPLEMENTARY FIGURES**

**
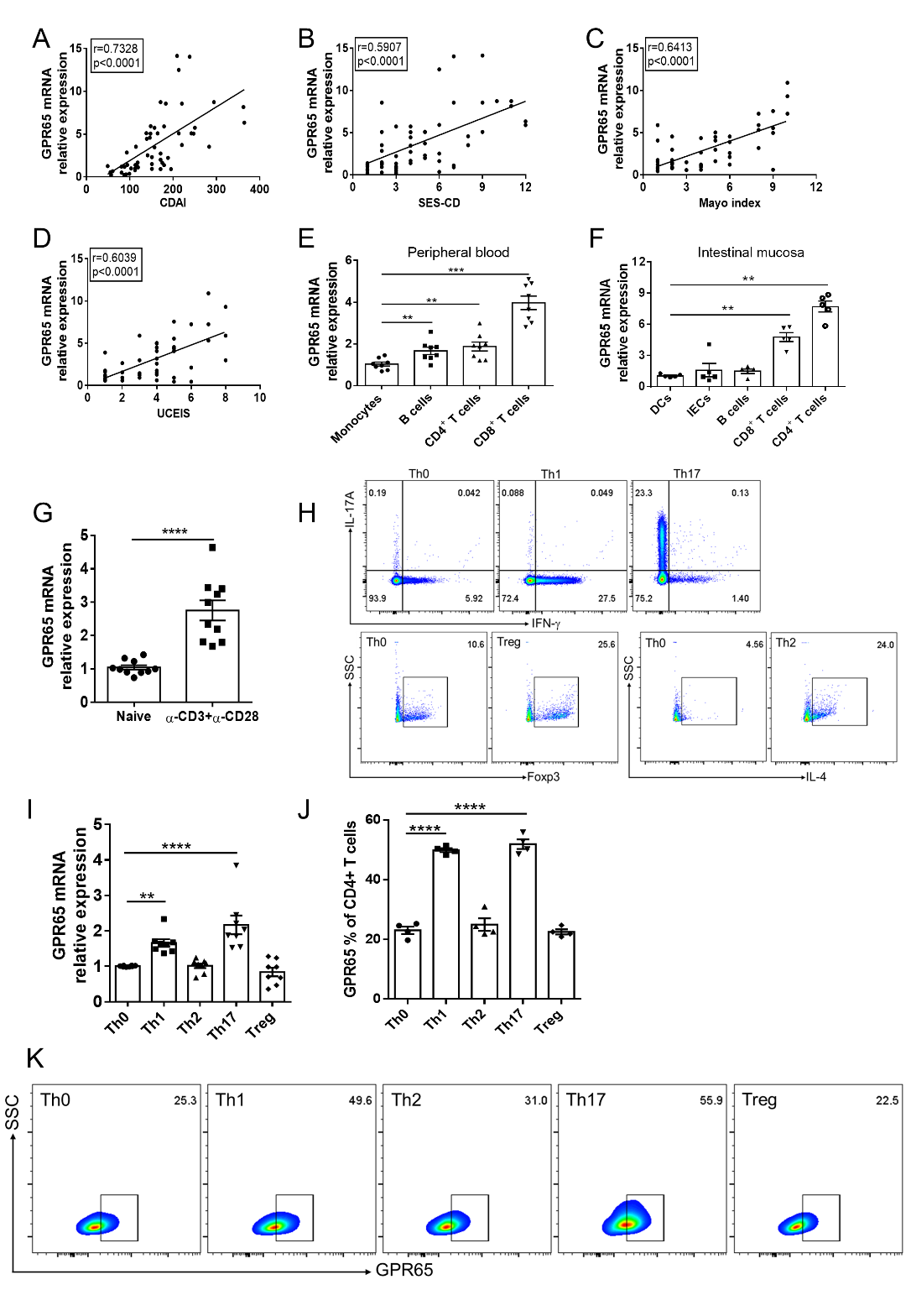
**

**Figure S1. GPR65 is upregulated in active IBD patients and refers to disease activities.** (**A** to **D**) GPR65 expression in intestinal mucosa of CD patients (n=56) was positively associated with CDAI (r=0.7328, p<0.0001 in **A**) and SES-CD (r=0.5907, p<0.0001 in **B**) and the correlation analysis between Mayo score (r=0.6413, p<0.0001 in **C**), UCEIS (r=0.6039, p<0.0001 in **D**) and GPR65 expression in intestinal mucosa of UC patients (n=49), respectively. (**E**) CD4^+^, CD8^+^ T, CD19^+^ B cells, and CD14^+^ monocytes were obtained from peripheral blood of 8 healthy donors by immunomagnetic positive selection, and expression of GPR65 was analyzed by qRT-PCR. (**F**) LP-CD4^+^, CD8^+^ T, CD19^+^ B cells, dendritic cells (DCs), and intestinal epithelial cells (IECs) were isolated from normal colon tissues of 5 patients who underwent colectomy for colon cancer, and the mRNA levels of GPR65 were analyzed by qRT-PCR. (**G**) PB-CD4^+^ T cells were separated from 8 healthy individuals and cultured with plate-bound anti-CD3 mAb (5 μg/mL) and soluble anti-CD28 mAb (2 μg/mL) for 48 hours, and qRT-PCR was performed to analyze GPR65 expression in these CD4^+^ T cells. (**H-I**) PB-CD4^+^ T cells were separated from 8 healthy individuals and cultured with plate-bound anti-CD3 mAb (5 μg/mL) and soluble anti-CD28 mAb (2 μg/mL) under Th0-, Th1-, Th2-, Th17- and Treg-polarizing conditions, respectively, for 5 days, the representative flow cytometry plot of differentiation efficiency was shown (**H)**, and qRT-PCR was performed to analyze GPR65 expression in these CD4^+^ T cells (**I**). (**J-K**) PB-CD4^+^ T cells were separated from 4 healthy individuals and cultured with plate-bound anti-CD3 mAb (5 μg/mL) and soluble anti-CD28 mAb (2 μg/mL) under Th0-, Th1-, Th2-, Th17- and Treg-polarizing conditions, respectively, for 5 days, and flow cytometry was performed to analyze GPR65 expression in these CD4^+^ T cells. Data were represented as mean ± SEM. Pearson's correlation was used (**A**, **B**, **C**, **D**), and unpaired Student’s *t* tests (**G**), ordinary one-way ANOVA followed by Dunnett's multiple comparison test was used (**E**, **F**, **I** and **J**). ***p*<0.01, ****p*<0.001.


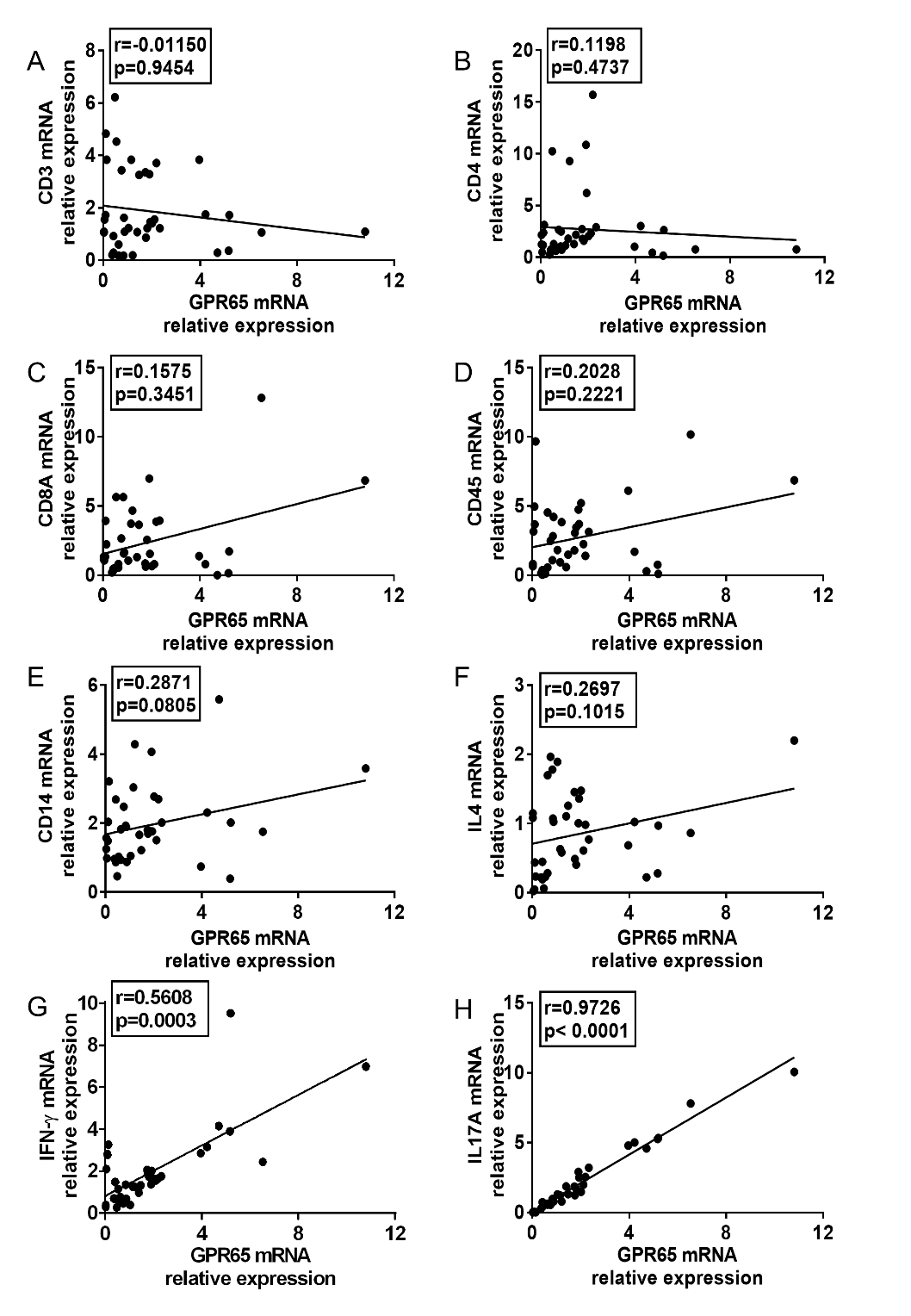


**Figure S2. GPR65 expression was positively correlated with the expression of IFN-γ and IL17A in intestinal mucosal tissues.** (**A**-**H**) Intestinal mucosal tissues were collected from 10 HC, 14 A-CD and 12 A-UC, and the expression of CD3, CD4, CD8, CD14, IL-4, IFN-γ, IL17A and GPR65 was detected by qRT-PCR. Pearson's correlation was used (**A** to **H**).


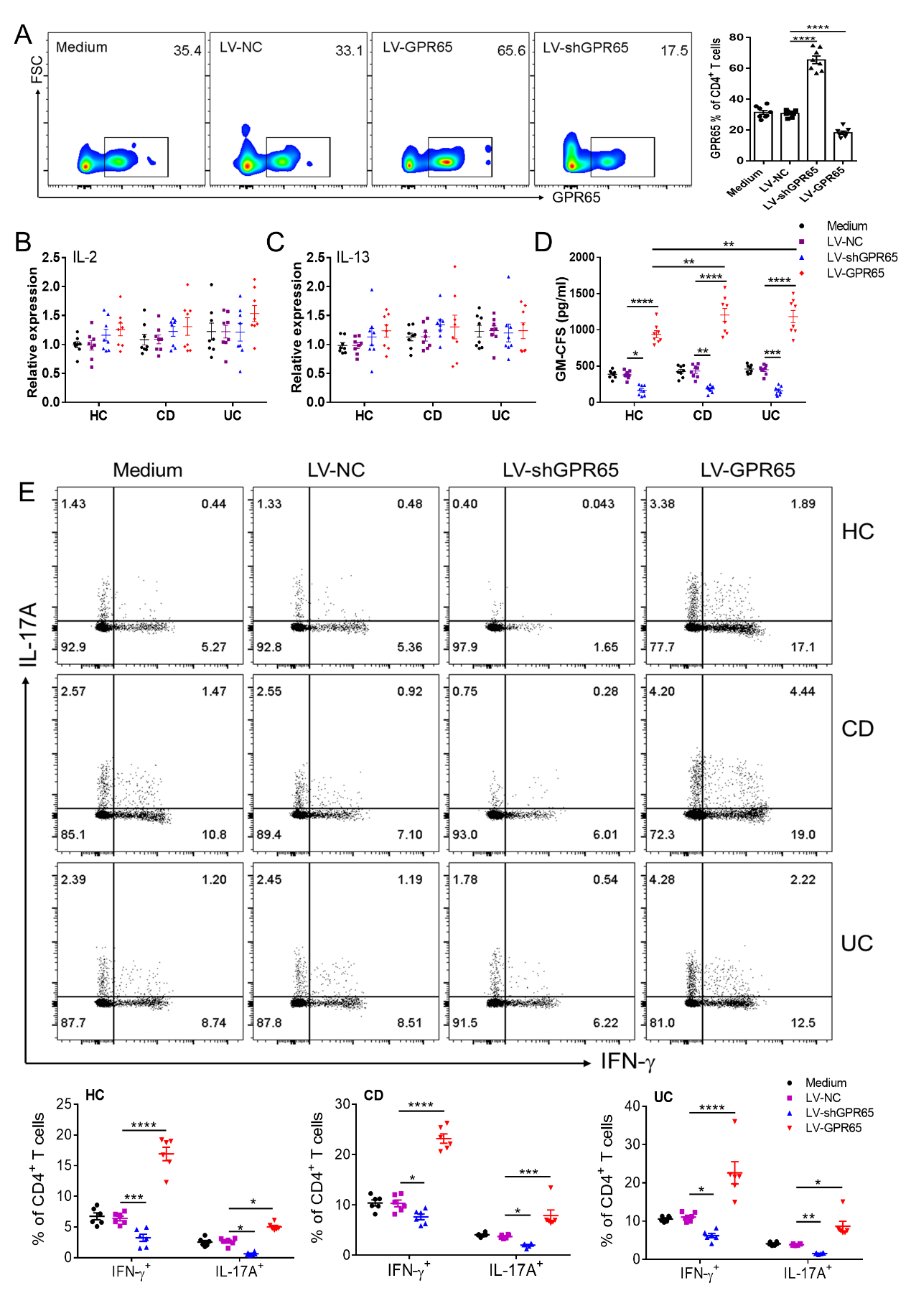


**Figure S3. Overexpression of GPR65 expression in CD4^+^ T cells promotes Th1/Th17 cell differentiation in IBD patients.** (**A** to **D**) PB-CD4^+^ T cells (1 × 10^5^/well) were isolated from 8 HC, 8 A-CD patients and 8 A-UC patients, then transfected with lentivirus expressing GPR65 shRNA (LV-shGPR65), GPR65 (LV-GPR65), and negative control (LN-NC), respectively, and cultured with plate-bound anti-CD3 mAb (5 μg/mL) and soluble anti-CD28 mAb (2 μg/mL) for 5 days. The protein level of GPR65 was determined by flow cytometry (**A**), IL-2 and IL-13 mRNA expression was performed by qRT-PCR (**B** and **C**), and the level of GM-CSF was detected in the culture suspension by ELISA in transfected CD4^+^ T cells (**D**). (**E**) PB-CD4^+^ T cells (1 × 10^5^/well) were isolated from 6 HC, 6 A-CD patients and 6 A-UC patients, then transfected with lentivirus expressing GPR65 shRNA (LV-shGPR65), GPR65 (LV-GPR65), and negative control (LN-NC), respectively, and cultured with plate-bound anti-CD3 mAb (5 μg/mL) and soluble anti-CD28 mAb (2 μg/mL) for 5 days. The frequencies of IL-17A and IFN-γ expression in transfected CD4^+^ T cells were analyzed by flow cytometry. Data were expressed as mean ± SEM. Statistical analysis was evaluated by Tukey's multiple comparison test. **p*<0.05, ***p*<0.01, ****p*<0.001, *****p*<0.0001.


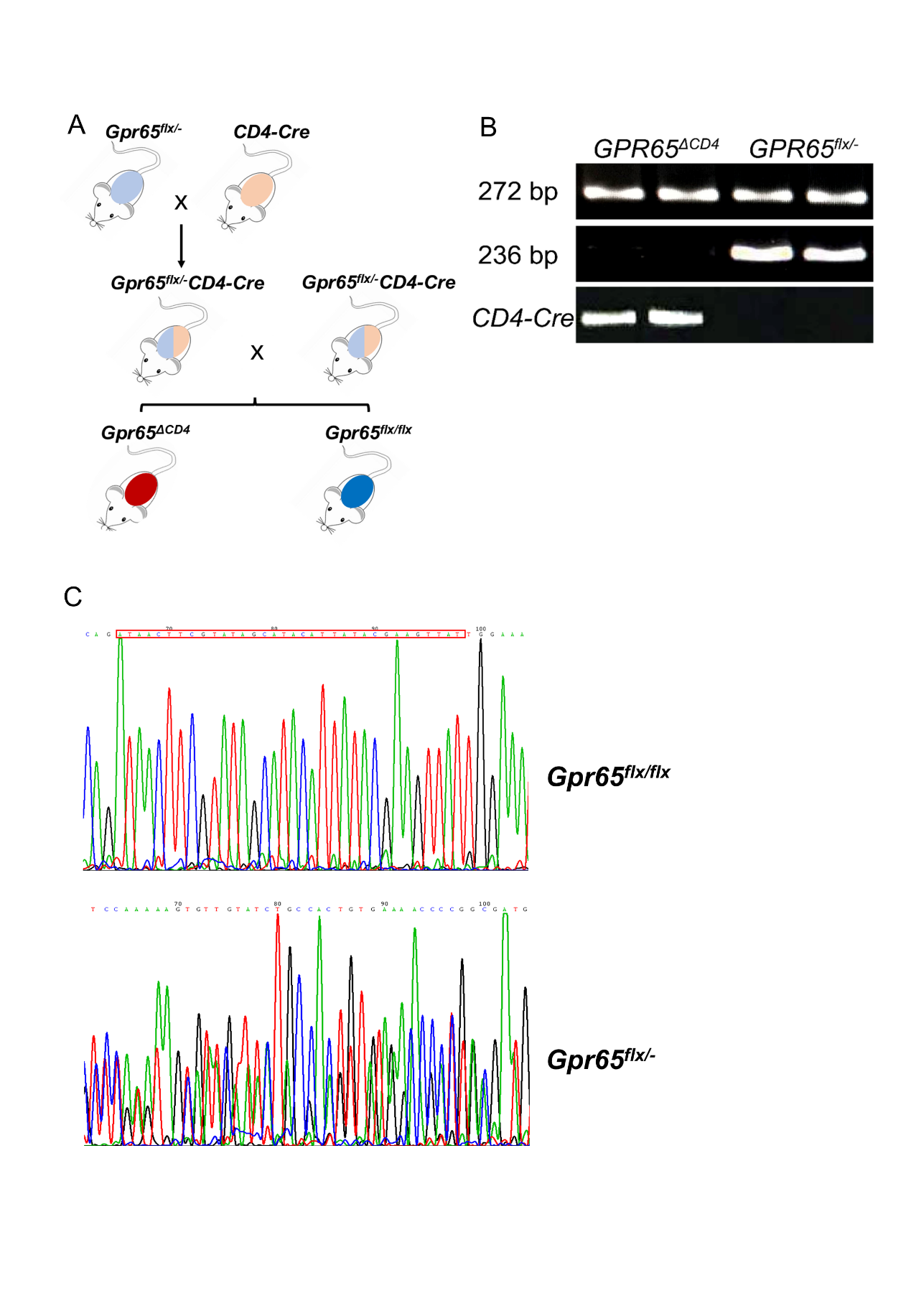


**Figure S4. The generation and genotype of *Gpr65^ΔCD4^* mice.** (**A**) *Gpr65^flx/-^* mice on a C57BL/6J background were crossed with *CD4-Cre* mice to generate *Gpr65^flx/-^CD4-Cre* mice as F1 progeny. Confirmed F1 heterozygotes were intercrossed to obtain *Gpr65^ΔCD4^* and *Gpr6^flx/flx^* mice. (**B**) Target loci of *Gpr65* were amplified using genomic DNA templates from mouse tails of *Gpr65^flx/-^* and *Gpr65^ΔCD4^* mice, and PCR products of targeted fragment were followed by agarose gel electrophoresis. (**C**) PCR products of targeted fragment were subjected to sequencing.


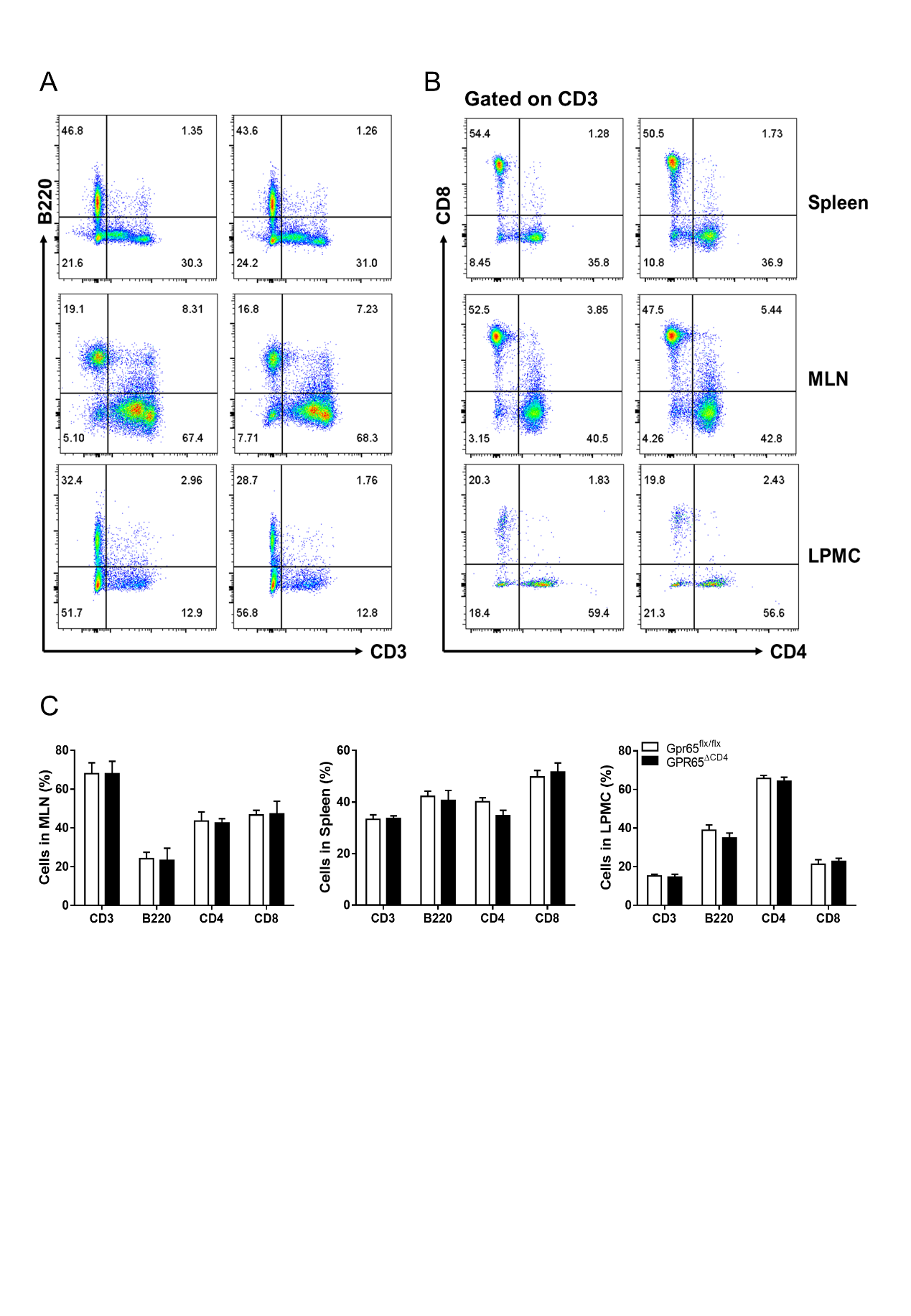


**Figure S5. Phenotypic analysis of B and T lymphocytes in spleen, MLN, and LPMC of** ***Gpr65^flx/flx^* and** ***Gpr65^ΔCD4^* mice.** (**A**) Single-cell suspension of spleen, MLN, and LPMC was obtained from 7-week-old *Gpr65^flx/flx^* and *Gpr65^ΔCD4^* mice (n=4/group), and stained with fluorochrome-conjugated anti-CD3, anti-CD4, anti-CD8 and anti-B220 mAbs, respectively. (**B**) The frequencies of B220^+^ B cells, CD3^+^ T cells (gated on total live cells), CD4^+^ and CD8^+^ T cells (gated on CD3^+^ T cells) in spleen, MLN, and LPMC were analyzed by flow cytometry. (**C**) Percentages of these cells in spleen, MLN, and LPMC were exhibited in the bar chart, respectively. Data were expressed as mean ± SEM. All statistical analyses were performed with Student’s unpaired *t* tests.


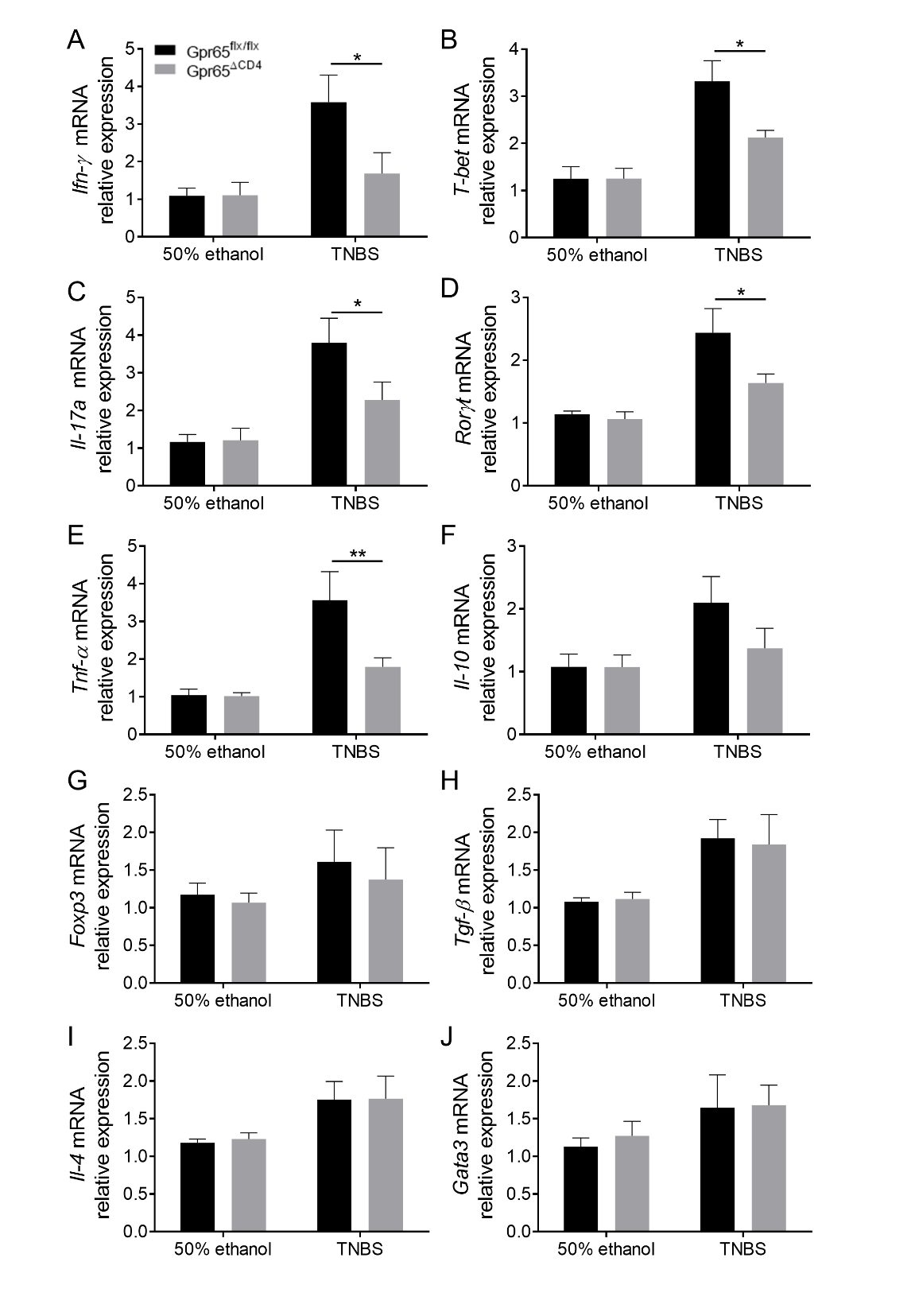


**Figure S6. Deficiency of GPR65 in CD4^+^ T cells ameliorates TNBS-induced acute colitis in mice.** TNBS-induced colitis model was established in in *Gpr65^ΔCD4^* and *Gpr65^flx/flx^* mice (n=10/group). (**A** to **J)** Colon tissues were collected from colitic mice and controls, and the mRNA levels of *Ifn-γ*, *T-bet*, *Il-17a*, *Rorγt*, *Tnf-α, Il-10*, *Foxp3, Tgf-β*, *Il-4*, and *Gata3* were analyzed by qRT-PCR. Data were expressed as mean ± SEM. Statistical analysis was evaluated using Student’s unpaired *t* test. **p*<0.05, *****p*<0.0001.


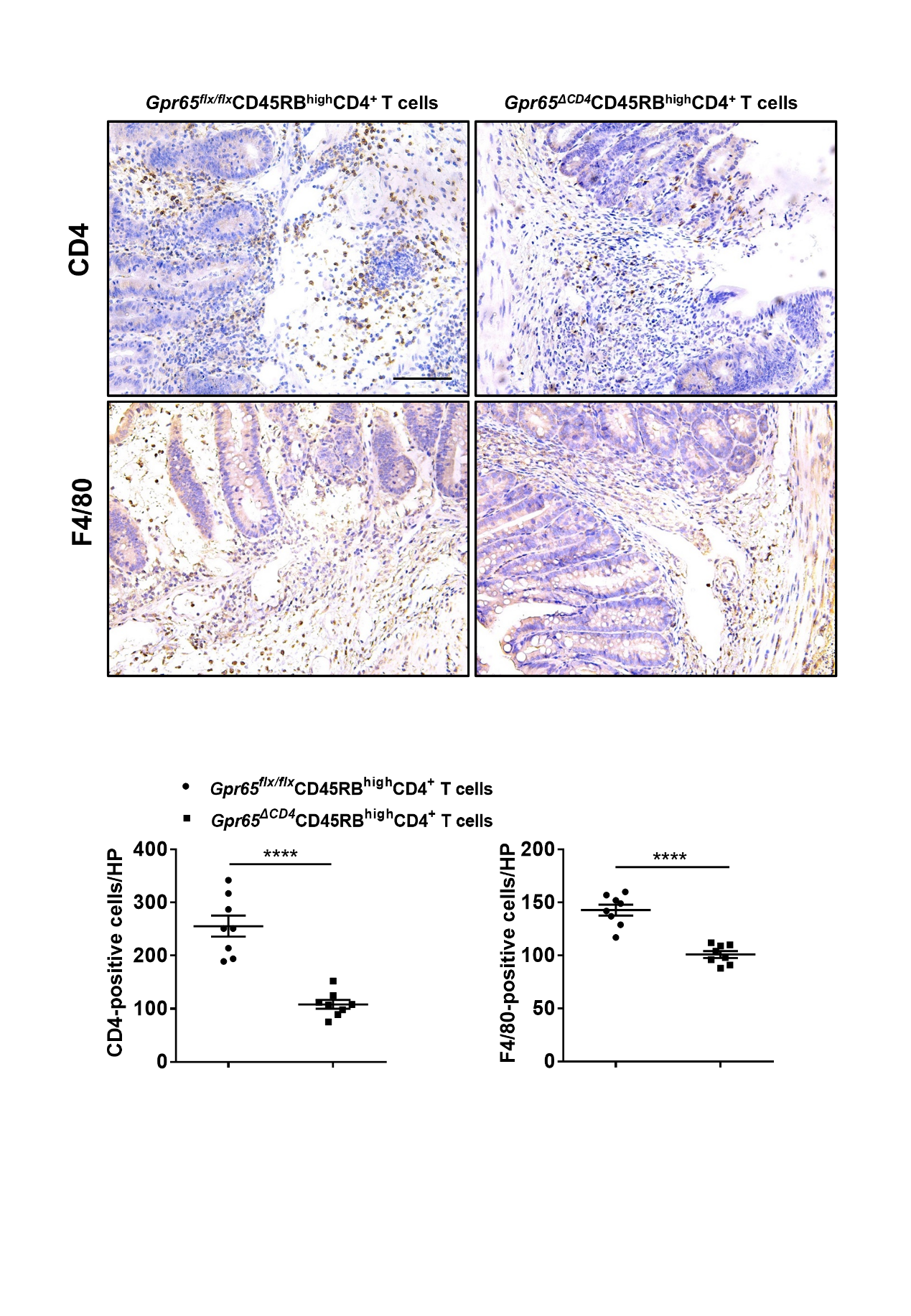


**Figure S7. The infiltration of CD4^+^ T cells and F4/80 macrophages in the colon tissues of chronic colitis model in Rag1^-/-^ mice.** Rag1^-/-^ mice were injected intraperitoneally with CD45RB^high^CD4^+^ T cells (5 × 10^5^ cells/mouse) from *Gpr65^flx/flx^* and *Gpr65^ΔCD4^* mice as indicated in **Fig 5**. Colon tissues were harvested to assess the levels of CD4^+^ T cell and F4/80^+^ macrophages in Rag1^-/-^ mice 7 weeks after T cell transfer by immunohistochemistry. Scale bar 100 μm. Data were expressed as mean ± SEM. Statistical analysis was evaluated using Student’s unpaired *t* test**.** *****p*<0.0001.

**
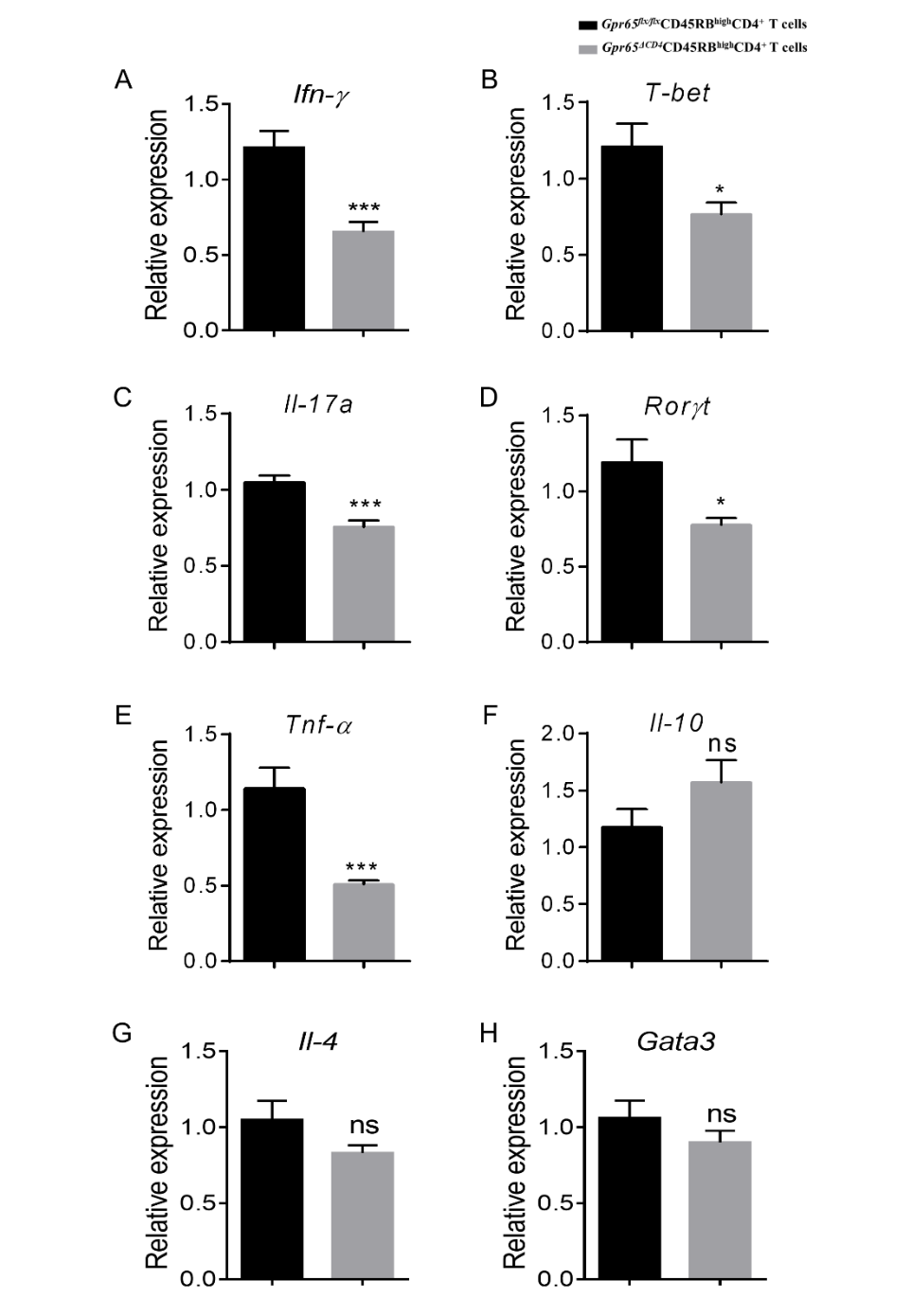
**

**Figure S8. *GPR65^ΔCD4^*CD45RB^high^CD4^+^ T cells mitigate intestinal mucosal chronic inflammation in Rag1^-/-^ mice.** Rag1^-/-^ mice were injected intraperitoneally with CD45RB^high^CD4^+^ T cells (5 × 10^5^ cells/mouse) from *Gpr65^flx/flx^* and *Gpr65^ΔCD4^* mice as indicated in **Fig 5**. (**A** to **H**) The mRNA levels of *Ifn-γ*, *T-bet*, *Tnf-α*, *Il-17a*, *Rorγt*, *Il-10*, *Il-4*, and *Gata3* in the colon tissues were assessed by qRT-PCR. Data were expressed as mean ± SEM. Statistical analysis was evaluated using Student’s unpaired *t* test. **p*<0.05, *****p*<0.0001.


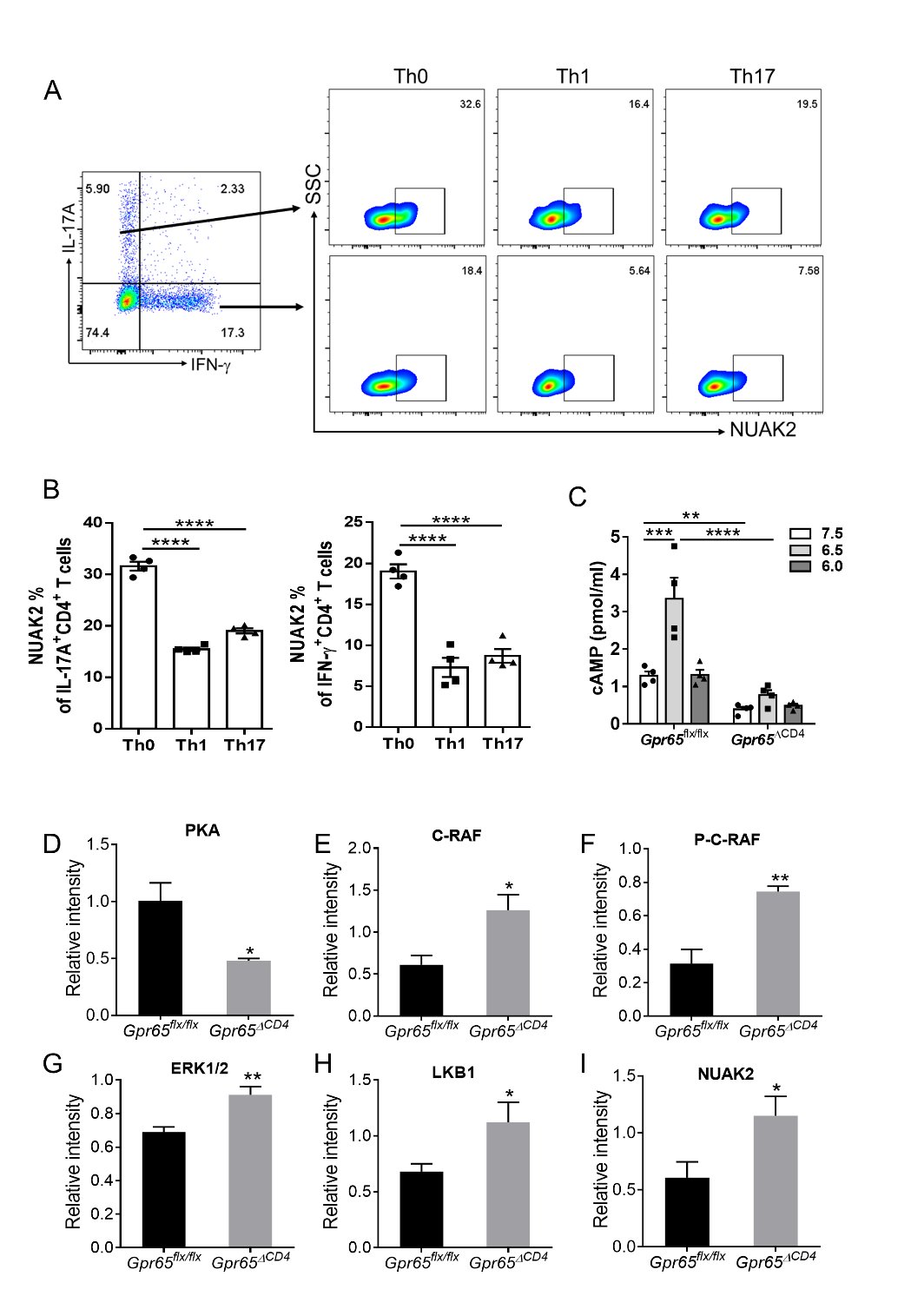


**Figure S9.** **GPR65 deficiency suppresses Th1 and Th17 cell immune response via activating C-Raf-EKR1/2-LKB1-NUAK2 pathway in CD4^+^ T cells.** PB-CD4^+^ T cells were isolated from 4 healthy controls, and cultured under different polarizing conditions. (**A**) These polarizing CD4^+^ T cells were harvested on day 5, and protein expression of IFN-γ, IL-17A and NUAK2 was evaluated by flow cytometry. (**B**) Percentages of IFN-γ^+^NUAK2^+^CD4^+^ T and IL-17A^+^NUAK2^+^CD4^+^ T cells were exhibited in the chart. (**C**) Splenic naïve CD4^+^ T cells were isolated from *Gpr65^flx/flx^* and *Gpr65^∆CD4^* mice (n=4/group), and culture in different pH (pH=7.5, 6.5 and 6) medium for 6 hours, and the culture supernatant was collected for cAMP detection by ELISA. (**D**-**I**) Relative intensity of PKA, C-Raf, P-C-Raf, ERK1/2, LKB1, and NUAK2 were detected in *Gpr65^flx/flx^* and *Gpr65^ΔCD4^* splenic CD4^+^ T cells by Image J. Data were expressed as mean ± SEM. Statistical analysis was evaluated using ordinary one-way ANOVA (**B** and **C**) and Student’s unpaired *t* test (**D**-**I**). **p*<0.05, ***p*<0.01, ****p*<0.001, *****p*<0.0001.


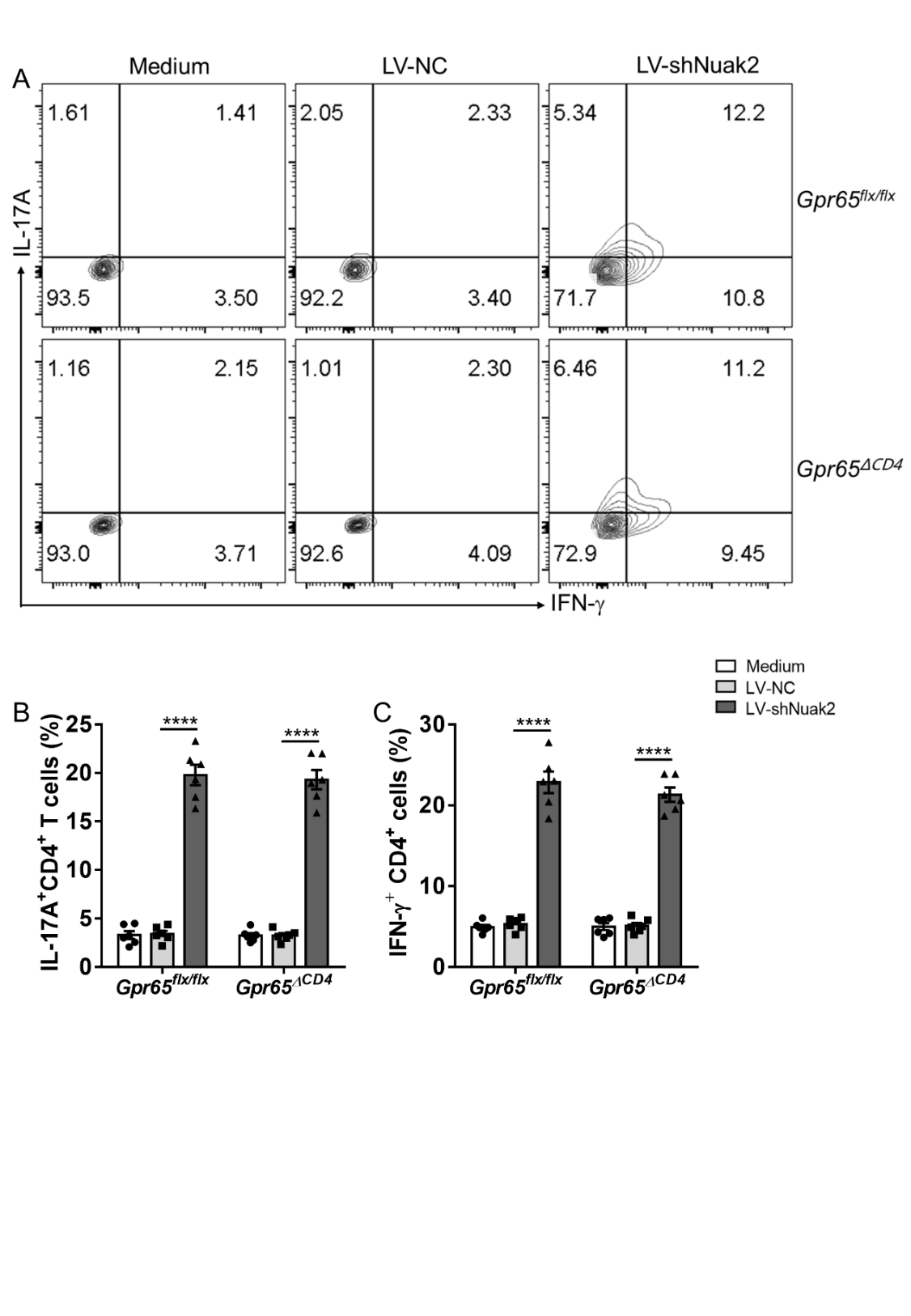


**Figure S10 Inhibition of Nuak2 expression in CD4^+^ T cells promotes Th1/Th17 cell differentiation.** Splenic naïve CD4^+^ T cells (1 × 10^5^/well) were isolated from Gpr65^flx/flx^ and Gpr65^ΔCD4^ mice (n=6/group), then transfected with lentivirus expressing Nuak2 shRNA (LV-shNuak2), and negative control (LN-NC), respectively, and cultured with plate-bound anti-CD3 mAb (5 μg/mL) and soluble anti-CD28 mAb (2 μg/mL) for 5 days. The frequencies of IL-17A and IFN-γ expression in transfected CD4^+^ T cells were analyzed by flow cytometry. Data were expressed as mean ± SEM. Statistical analysis was evaluated multiple *t* test. *****p*<0.0001.


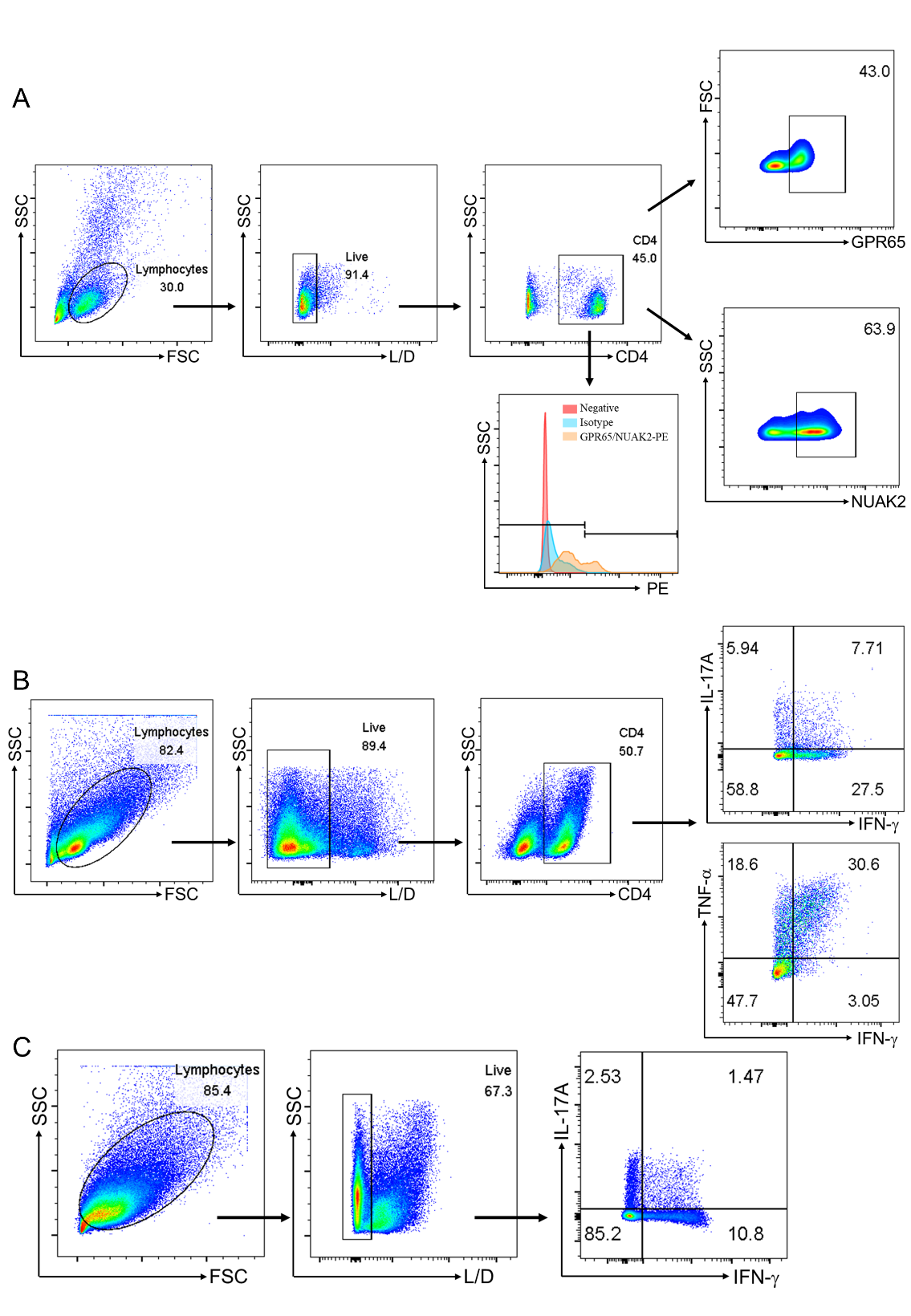


**Figure S11. Flow cytometry gating strategies.** (**A**) Gating strategies for human PB-CD4^+^ T cells used in Figure 1G and 7H. (**B**) Gating strategies for mouse CD4^+^ T cells used in Figure 4G-H and 5G. (**C**) Gating strategies for human or mouse CD4^+^ T cells used in Figure 3A, S3E, S9A, and S10A.

**SUPPLEMENTARY METHODS**

**Flow cytometry**

For cell surface staining, the LPMCs and single cell suspensions of spleens of Gpr65^flx/flx^ and Gpr65^ΔCD4^ mice were obtained and incubated with Fc Block (BD). These cells were then incubated with the Live/Dead Fixable Dead Cell stain kits (Invitrogen) and fluorochrome-conjugated anti-CD4 and CD45RB mAbs or isotype (1:100, Biolegend) for 30 mins on ice. For intracellular cytokine staining, PB-CD4^+^ T cells were fixed and permeabilized on ice for 30 mins, and then incubated with anti-GPR65 (1:100, Alomone labs), anti-NUAK2 (1:100, Invitrogen) or isotype for 30 mins on ice. After 3 washes, these samples were stained with anti-rabbit IgG-PE (1:1000, Invitrogen) for 30 mins on ice. Additionally, LPMCs were treated with phorbol 12-myristate 13-acetate (PMA) (50 ng/mL, Sigma-Aldrich), ionomycin (750 ng/mL, Sigma-Aldrich) and brefeldin A (3 μg/mL, eBioscience) for 5 hours at 37°C. Subsequently, these cells were surface-stained, fixed and permeabilized on ice for 30 mins followed by washing, and intracellular staining was carried out with fluorochrome-conjugated anti-IFN-γ, IL-17A, and TNF-α mAbs (1:100, Biolegend), respectively. All stained samples were analyzed on a BD FACSCanto II Flow Cytometer. Data were processed using FlowJo software (Tree Star; Ashland, OR).

**Th cell differentiation *in vitro***

Naïve splenic CD4^+^ T cells were isolated from Gpr65^ΔCD4^ and Gpr65^flx/flx^ mice by using anti-mouse naive CD4^+^ T cell isolation kit (Miltenyi Biotecanti). Naïve PB-CD4^+^ T cells were isolated from healthy controls by using anti-human naive CD4^+^ T cell enrichment set (BD). These CD4^+^ T cells (5×10^5^/well) were activated with plate-coated anti-CD3 mAb (5 μg/mL, eBioscience) and soluble anti-CD28 mAb (2 μg/μL, eBioscience) in complete RPMI 1640 medium, and induced to differentiate into Th1 cells by supplementation with IL-12 (10 ng/mL, R&D) plus anti-IL-4 antibody (10 μg/mL, eBioscience); Th17 cells with IL-1β (10 ng/mL, R&D), IL-6 (30 ng/mL, R&D), IL-23 (20 ng/mL, R&D) plus anti-IL-4 antibody (10 μg/mL, eBioscience) and anti-IFN-γ antibody (10 μg/mL, eBioscience); Th2 cells with IL-4 (50 ng/ml, R&D) plus anti-IFN-γ antibody (10 μg/mL, eBioscience); and Tregs with TGF-β (10 ng/ml, R&D) plus anti-IL-4 antibody (10 μg/mL, eBioscience) and anti-IFN-γ antibody (10 μg/mL, eBioscience), respectively. After 5 days, they were harvested and then stimulated with phorbol 12-myristate 13-acetate (50 ng/mL, Sigma), ionomycin (1000 ng/mL, Sigma) and brefeldin A (3 μg/mL, Biolegend) for 5 hours. Intracellular expression of IL-17A and IFN-γ was analyzed by flow cytometry (BD).
