# Supplementary material for: GPR65 promotes intestinal mucosal Th1 and Th17 cell differentiation and gut inflammation through downregulating NUAK2
Source: Clin Transl Med. 2022 Mar 28;12(3):e771. doi: 10.1002/ctm2.771 (PMC8958354; doi:10.1002/ctm2.771)
Supplement: Supplementary file 2 — Supporting information [file CTM2-12-e771-s002.docx]

| **Supplementary Table 1. Clinical characteristics of IBD patients and healthy controls** | | | | | | | |
| --- | --- | --- | --- | --- | --- | --- | --- |
|  | **Biopsy samples** | | |  |  | **Blood samples** |  |
|  | **HCs** | **Patients**  **with CD (A/R)^b^** | **Patients**  **with UC (A/R)^b^** |  | **HCs** | **Patients**  **with CD (A/R)^b^** | **Patients**  **with UC (A/R)^b^** |
|  |  |  |  |  |  |  |  |
| **Number of patients** | 64 | 118 (87/31) | 112 (77/35) |  | 46 | 84 (46/38) | 71 (50/21) |
| **Gender** |  |  |  |  |  |  |  |
| Male | 31 | 65 (47/18) | 52 (36/16) |  | 25 | 38 (20/18) | 39 (28/11) |
| Female | 33 | 53 (40/13) | 60 (41/19) |  | 21 | 46 (26/20) | 32 (22/10) |
| **Age (years)** | 31.5 ± 7.8 | 38.7 ± 10.4 | 39.8 ± 14.3 |  | 33.7 ± 9.3 | 34.4 ± 7.5 | 35.7 ± 8.6 |
| **Disease duration (months)** |  | 46.9 ± 18.2 | 48.6 ± 20.1 |  |  | 38.5 ± 14.8 | 40.8 ± 14.9 |
| **Disease location (CD)^a^** |  |  |  |  |  |  |  |
| L1 |  | 39 (28/11) |  |  |  | 13 (8/5) |  |
| L2 |  | 22 (15/7) |  |  |  | 29 (11/9) |  |
| L3 |  | 57 (44/13) |  |  |  | 53 (27/24) |  |
| L4 |  | 0 |  |  |  | 0 |  |
| **Disease extent (UC)^a^** |  |  |  |  |  |  |  |
| E1 |  |  | 28 (16/12) |  |  |  | 17 (9/8) |
| E2 |  |  | 40 (29/11) |  |  |  | 25 (17/8) |
| E3 |  |  | 44 (32/12) |  |  |  | 29 (24/5) |
| **Current therapy** |  |  | |  | |  | |
| 5-Aminosalicylates |  | 22 (14/8） | 46 (25/21) |  |  | 19 (12/7) | 26 (16/10) |
| Biologics |  | 72 (51/21) | 29 (21/8) |  |  | 41 (23/20) | 17 (11/6) |
| Azathioprine |  | 28 (18/10) | 28 (18/10) |  |  | 19 (13/6) | 23 (15/8) |
| Methotrexate |  | 8 (6/2) | 0 |  |  | 9 (6/3) | 0 |
| Glucocorticoids |  | 21 (21/0) | 40 (29/11) |  |  | 13 (11/2) | 27 (20/7) |
| **CDAI/Mayo Score (Mean±SEM)** |  | 231.2 ± 41.4  /98.7 ± 22.5 | 7.2±1.6  /1.4±0.5 |  |  | 221.3 ± 38.2  /91.7 ± 19.4 | 6.3±1.9  /1.2±0.6 |
|  |  |  |  |  |  |  |  |
| ^a^According to the Montreal classification system. | | | | | | | |
| ^b^A/R, Active/Remission. | | | | | | | |

**Supplementary Table 2. The primer sequences [5’ to 3’] used in qRT-PCR**

| **Gene** |  | **Forward Primer** | **Reverse Primer** |
| --- | --- | --- | --- |
| Human | GPR65 | ACAGCAATTCTGGGAAGCGA | ATTACACCTCCCAGTGCAGAA |
|  | IL-17A | TCCCACGAAATCCAGGATGC | GGATGTTCAGGTTGACCATCAC |
|  | RORC | GTGGGGACAAGTCGTCTGG | AGTGCTGGCATCGGTTTCG |
|  | IFN-γ | TCACCGTCCTAGAAGGATTCAG | AAACTCTGGTGGTTCAAAAGACA |
|  | T-bet | TTGAGGTGAACGACGGAGAG | CCAAGGAATTGACAGTTGGGT |
|  | TNF-α | GAGGCCAAGCCCTGGTATG | CGGGCCGATTGATCTCAGC |
|  | GATA3 | GCCCCTCATTAAGCCCAAG | TTGTGGTGGTCTGACAGTTCG |
|  | IL-4 | CGGCAACTTTGTCCACGGA | TCTGTTACGGTCAACTCGGTG |
|  | IL-10 | GACTTTAAGGGTTACCTGGGTTG | TCACATGCGCCTTGATGTCTG |
|  | FOXP3 | GTGGCCCGGATGTGAGAAG | GGAGCCCTTGTCGGATGATG |
|  | NUAK2 | GATGCACATACGGAGGGAGAT | GCTGGCATACTCCATGACGAT |
| Mouse | Gpr65 | CGCCACCTCGAACACTATTTG | GCATAAAGATCCGATGTTGGCT |
|  | Il-17a | TCAGCGTGTCCAAACACTGAG | CGCCAAGGGAGTTAAAGACTT |
|  | Rorγt | CGCGGAGCAGACACACTTA | CCCTGGACCTCTGTTTTGGC |
|  | Ifn-γ | ATGAACGCTACACACTGCATC | CCATCCTTTTGCCAGTTCCTC |
|  | T-bet | AGCAAGGACGGCGAATGTT | GTGGACATATAAGCGGTTCCC |
|  | Tnf-α | CAGGCGGTGCCTATGTCTC | CGATCACCCCGAAGTTCAGTAG |
|  | Gata3 | CTCGGCCATTCGTACATGGAA | GGATACCTCTGCACCGTAGC |
|  | Il-4 | GGTCTCAACCCCCAGCTAGT | GCCGATGATCTCTCTCAAGTGAT |
|  | Il-10 | AGCCTTATCGGAAATGATCCAGT | GGCCTTGTAGACACCTTGGT |
|  | Foxp3 | CACCTATGCCACCCTTATCCG | CATGCGAGTAAACCAATGGTAGA |
|  | Tgf-β | CCACCTGCAAGACCATCGAC | CTGGCGAGCCTTAGTTTGGAC |
|  | Nuak2 | CTACGCCTCGCCTGAGATAGT | TCACCAGTGTTTTATGATCCTGC |

**Supplementary Table 3. Reagents**

| **Reagent** | **Sources** | **Identifier** |
| --- | --- | --- |
| **Antibodies** |  |  |
| Anti-GPR65 | Alomone labs | AGR-043 |
| Anti-GPR65-PE | Alomone labs | AGR-043-PE |
| Anti-human IL-17A-PE-Cy7 | Biolegend | 512315 |
| Anti-human IFN-γ-PerCP-Cy5.5 | Biolegend | 502526 |
| Anti-human CD4-PE-Cy7 | Biolegend | 357410 |
| Anti-human CD4-FITC | Biolegend | 317408 |
| Anti-mouse B220-PE-Cy7 | Biolegend | 103222 |
| Anti-mouse CD3-APC | Biolegend | 100236 |
| Anti-mouse CD8-PE | Biolegend | 100708 |
| Anti-mouse CD4-FITC | Biolegend | 100406 |
| Anti-mouse CD4-PerCP-Cy5.5 | Biolegend | 100434 |
| Anti-mouse IL-17A-PE | Biolegend | 506904 |
| Anti-mouse IL-17A-PE-Cy7 | Biolegend | 506922 |
| Anti-mouse IFN-γ-FITC | Biolegend | 505806 |
| Anti-mouse IFN-γ-PerCP-Cy5.5 | Biolegend | 505822 |
| Anti-mouse TNF-α-APC | Biolegend | 506308 |
| Anti-CD4 | Santa Cruz | sc-13573 |
| Anti-F4/80 | Thermofisher | MA1-91124 |
| Anti-NUAK2 | Thermofisher | PA582371 |
| Anti-PKA | Cell Signaling Technology | 4782S |
| Anti-CRAF | Cell Signaling Technology | 9421T |
| Anti-P-CRAF | Cell Signaling Technology | 9422T |
| Anti-ERK1/2 | Thermofisher | 13-6200 |
| Anti-LKB1 | Cell Signaling Technology | 3047S |
| Anti-ACTIN | Santa Cruz | sc-8432 |
| Rabbit IgG Isotype Control | Thermofisher | 02-6102 |
| Donkey anti-Rabbit IgG Secondary Antibody, PE | Thermofisher | 12-4739-81 |
| Alexa fluor® 488 conjugated goat anti-rat IgG | Thermofisher | A-11006 |
| Alexa fluor® 594 conjugated donkey anti-rabbit IgG | Thermofisher | R-37119 |
| Alexa fluor® 488 conjugated donkey anti-rabbit IgG | Thermofisher | A-11029 |
| **ELISA kit** |  |  |
| HUMAN IL-17A | Biolegend | 433914 |
| HUMAN IFN-γ | Biolegend | 430101 |
| HUMAN TNF-α | Biolegend | 430201 |
| HUMAN-IL-10 | Biolegend | 430601 |
| HUMAN-IL-4 | Biolegend | 430301 |
| Direct cAMP ELISA kit | Enzo Life Sciences | ADI-900-066A |
| GM-CSF | Biolegend | 432001 |
| **Other Reagents** |  |  |
| Human Naive CD4 T Cell Enrichment Set | BD | 558521 |
| Naive CD4+ T Cell Isolation Kit, mouse | Miltenyi Biotec | 130-104-453 |
| Anti-Human CD19 Magnetic Particles | BD | 551520 |
| Anti-Human CD8 Magnetic Particles | BD | 557766 |
| Anti-Human CD14 Magnetic Particles | BD | 557769 |
| Human Dendritic Cell Enrichment Set | BD | 558420 |
| Anti-Human CD4 Particles | BD | 557767 |
| Anti-Mo IFN-γ | Thermofisher | 16-7311-85 |
| Anti-Mo CD3 | Thermofisher | 16-0032-85 |
| Anti-Mo CD28 | Thermofisher | 16-0281-85 |
| Anti-Mo IL4 | Thermofisher | 16-7041-85 |
| Anti-Hu IFN-γ | Thermofisher | 16-7318-85 |
| Anti-Hu CD3 | Thermofisher | 16-0037-85 |
| Anti-Hu CD28 | Thermofisher | 16-0289-85 |
| Anti-Hu IL4 | Thermofisher | 16-7048-85 |
